# Synthesis and applications of carbon nanomaterials for energy generation and storage

**DOI:** 10.3762/bjnano.7.17

**Published:** 2016-02-01

**Authors:** Marco Notarianni, Jinzhang Liu, Kristy Vernon, Nunzio Motta

**Affiliations:** 1 Institute of Future Environments and School of Chemistry, Physics, and Mechanical Engineering, Queensland University of Technology, Brisbane QLD 4001, Australia; 2 Plasma-Therm LLC, 10050 16th St. North, St. Petersburg, FL 33716, USA,; 3 School of Materials Science and Engineering, Beihang University, Beijing 100191, China

**Keywords:** carbon, carbon nanotubes, current collector, energy, fullerenes, gold nanoparticles, graphene, nanomaterials, organic solar cells, plasmonic structures, supercapacitors, thin films

## Abstract

The world is facing an energy crisis due to exponential population growth and limited availability of fossil fuels. Over the last 20 years, carbon, one of the most abundant materials found on earth, and its allotrope forms such as fullerenes, carbon nanotubes and graphene have been proposed as sources of energy generation and storage because of their extraordinary properties and ease of production. Various approaches for the synthesis and incorporation of carbon nanomaterials in organic photovoltaics and supercapacitors have been reviewed and discussed in this work, highlighting their benefits as compared to other materials commonly used in these devices. The use of fullerenes, carbon nanotubes and graphene in organic photovoltaics and supercapacitors is described in detail, explaining how their remarkable properties can enhance the efficiency of solar cells and energy storage in supercapacitors. Fullerenes, carbon nanotubes and graphene have all been included in solar cells with interesting results, although a number of problems are still to be overcome in order to achieve high efficiency and stability. However, the flexibility and the low cost of these materials provide the opportunity for many applications such as wearable and disposable electronics or mobile charging. The application of carbon nanotubes and graphene to supercapacitors is also discussed and reviewed in this work. Carbon nanotubes, in combination with graphene, can create a more porous film with extraordinary capacitive performance, paving the way to many practical applications from mobile phones to electric cars. In conclusion, we show that carbon nanomaterials, developed by inexpensive synthesis and process methods such as printing and roll-to-roll techniques, are ideal for the development of flexible devices for energy generation and storage – the key to the portable electronics of the future.

## Review

### The energy future: challenges and opportunities

The demand for energy in the 21st century is increasing due to the increase in the world’s population and rapid technological advancement [1]. Today, the worldwide population is using about 17 trillion watts of power with around 4 trillion watts being consumed in the United States alone [2]. Energy experts are predicting that we will need an additional 30 trillion watts by 2050 due to the global population growth and worldwide economic development [3].

Solving this energy demand using more efficient or clean alternative energy sources will not only save the planet from harmful effects caused by pollution but could also reduce disparity and create a more peaceful world [4]. Energy is just one of the many problems that the world is facing but it is probably the most important to be addressed with urgency in order to also solve other offshoot problems. In one of his last talks, Richard E. Smalley, the 1996 Nobel Laureate in Chemistry for the discovery of the fullerene, presented a list named “Top Ten Problems of Humanity for the Next 50 Years” [5]. The list was presented in order of priority as:

1. Energy
2. Water
3. Food
4. Environment
5. Poverty
6. Terrorism and war
7. Disease
8. Education
9. Democracy
10. Population

The energy problem is on the top of the list because, according to Smalley, it directly influences the other problems and thus should be prioritized accordingly by governments worldwide. The first immediate solution to this problem would be to work on energy efficiency programs. With the implementation of such programs, it has been demonstrated that developed nations could already reduce energy consumption by 25% [6].

**Figure 1:**
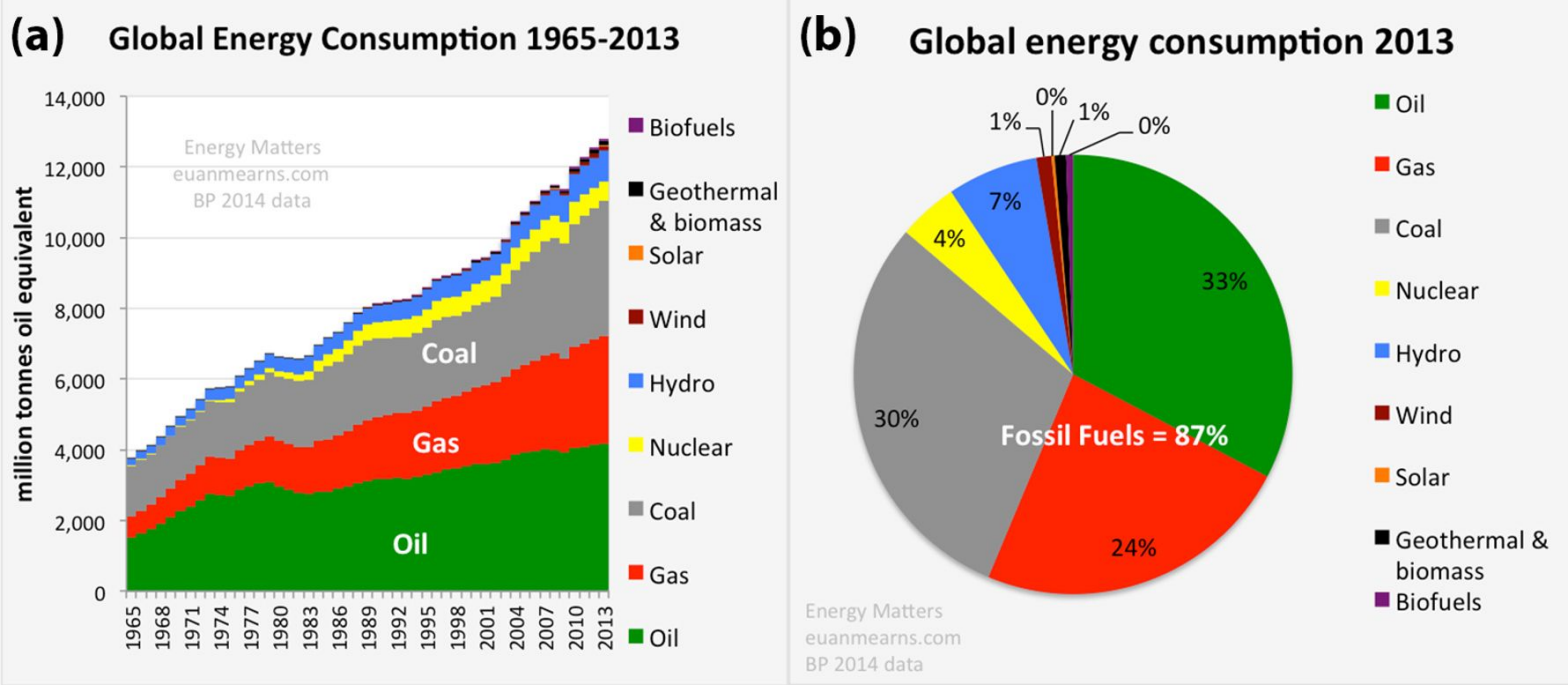
(a) Global energy consumption growth from 1965 to 2013. (b) The share of different energy sources for global energy consumption in 2013 [7].

The majority (about 87%) of energy produced in 2013 was composed of fossil fuels such as oil, gas and coal, which represented the best choice of energy production at competitive costs in the 20th and 21st century (Figure 1) [7]. Unfortunately, It has been proven that fossil fuels have catastrophic consequences for human health [8] and global warming [9] and their reserves are progressively decreasing [10].

**Figure 2:**
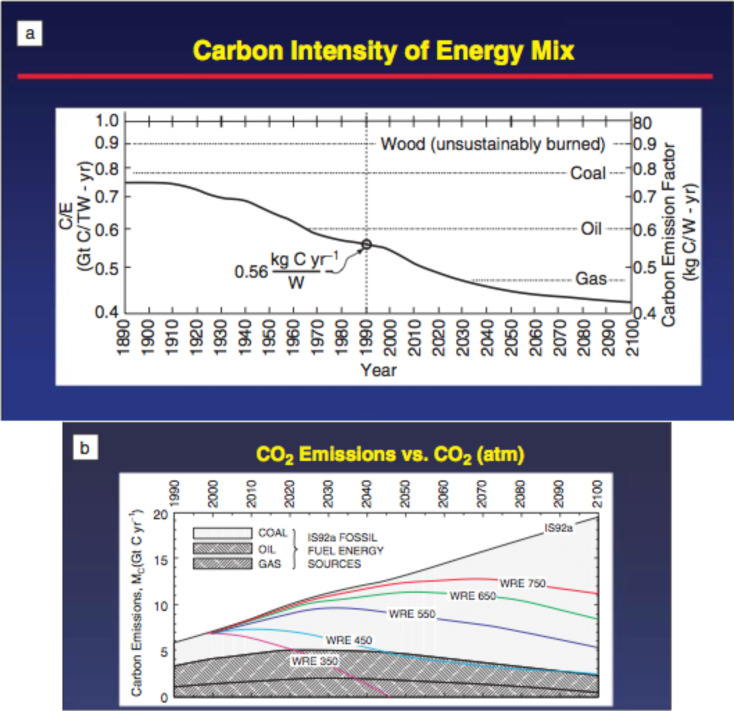
(a) Carbon concentration in the energy mixture from 1890–2100 (projected), i.e., kilograms of carbon emitted into the atmosphere as CO_2_ per year per watt of power, produced from combined sources of fuel. The average in 1990 is shown as an open circle on the carbon intensity curve. Reprinted with permission from [11]. Copyright (1998) Nature Publishing Group. (b) CO_2_ emissions versus CO_2_ in the atmosphere, projected through 2100. Reprinted with permission from [12]. Copyright (2011) Cambridge University Press.

Despite the fact that technological advances are able to reduce the amount of kilograms of carbon emitted into the atmosphere as CO_2_ per year per watt (Figure 2a), CO_2_ emissions continue to increase due to the increase in worldwide energy consumption (Figure 2b) [11]. For example, to stabilize the concentration of CO_2_ at 350 ppm (purple line in Figure 2b), ideally, we will need to reduce worldwide carbon emissions to zero by 2050 [12].

It has been estimated that in order to generate about 1/3 of the prospective energy needed by 2050, we should build around 10,000 nuclear plants over the next 36 years [12]! Apart from the costs of building these nuclear plants, nuclear energy has associated risks and hazards. Nuclear plants are in fact very expensive to build, maintain and protect from attack. Not to mention that the disposal of nuclear waste has still not been resolved, leaving problems for future generations to deal with [13].

Renewable energy represents the easiest way to produce clean and safe energy but included only 10% of the resources used in 2013 (Figure 1). Unfortunately, the cost of producing energy from renewable sources is still high. However, costs are decreasing, allowing these technologies to be considered in the near future when the price of fossil fuels increase due to their scarcity. Among all of the renewable energy resources available, including hydroelectricity, geothermal energy, wind energy, biomass and others, solar energy likely represents the best renewable resource. In fact, the biggest nuclear reactor that we can even imagine is the sun, which has provided energy to the earth for over 4 billion years and provides more energy in one hour than all of the energy consumed on our planet in an entire year. On Earth, we receive about 170,000 trillion watts of electromagnetic radiation. Therefore, covering 0.16% of the land on earth with 10% efficient solar conversion systems would provide over 20 trillion watts of power [3]. However, apart from the costs of this technology at the moment ($0.20–0.50 per kW·h), building solar farms in remote areas is not without problems. In fact, the advantage of oil in the last century (and still today) is its transportability across oceans without the need to build expensive infrastructure. For example, the price per gallon of gas includes less than 10% of the transportation cost to the gas station [12]. On the other hand, the cost of building solar farms is very high when infrastructure is factored into the transport of the electricity from remote desert areas to the urban centers where 25% of energy is lost in the transportation [3]. Receiving incentives from the government to install photovoltaic systems on private property could be a viable solution to immediately benefit from the energy produced without the construction of additional infrastructure and without transportation losses. However, this could definitely create a sort of energy independence that is not favored by major energy corporations. The energy business is one of the biggest in the world including companies such as Exxon Mobil (which was listed second in the United States in 2014 for publicly traded companies as having the greatest market capitalization) and Saudi Aramco (an oil state owned company), estimated from $781 billion to $7 trillion [14].

The other major problems of solar energy are that it is diffuse (170 W/m^2^) and intermittent. This is why concentration and storage become two critical issues to solve in order to make this energy source cost competitive with fossil fuels. The challenge over the next few years will not just be to produce electricity in a safe and clean way but also how to store the energy produced using technologies more efficient and more environmentally friendly than chemical batteries [13]. Creating small-scale energy storage technologies combined with smart grid technologies could help to provide energy to individual households when immediately needed and with a high efficiency.

Nanotechnology could help to solve solar energy’s obstacles and meet energy expectations without compromising the environment and human health by creating new devices that are able to generate, store and transport electricity in a clean and more efficient way and with smaller space requirements. Specifically, the use of nanostructured allotrope forms of carbon and derivatives such as fullerenes, carbon nanotubes (CNTs) and graphene have been widely investigated over the past 10 years for energy generation and storage. In particular, the possibility to include these nanostructured materials using lightweight flexible substrates, printable inks, low temperature and ambient pressure fabrication tools allows for a dramatic reduction in production costs [15].

Organic solar cell devices and electrochemical capacitors, also called supercapacitors, based on carbon nanostructures could allow for the fabrication of devices in the near future that could be more efficient and cheaper to produce than conventional silicon solar cells and chemical batteries [16–17]. The potential to produce these devices “in house” with simple tools such as printers, scissors and glue makes these technologies widely accessible, including in developing countries.

### Carbon nanomaterials: properties and synthesis

**Figure 3:**
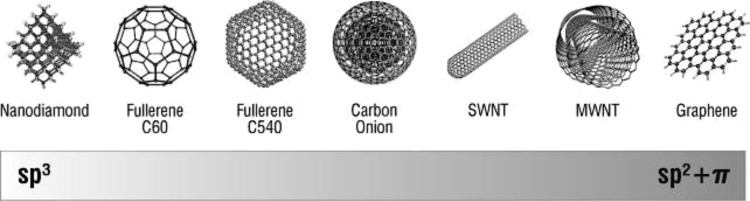
Hybridization states of carbon-based nanomaterials. Reprinted with permission from [19]. Copyright (2008) American Chemical Society.

Carbon, one of the most abundant materials found on earth, can be found in nature in its elemental form as graphite, diamond and coal. Its production is about 9 Gt/year for technological applications, constituting the highest production compared to all other elements [18]. Nanostructured allotrope forms of carbon have been intensively investigated in the past two decades because of their unique hybridization properties and sensitivity to perturbation during synthesis, allowing for fine manipulation of the material properties. In particular, carbon can be found in several different hybridization states, each having unique properties (Figure 3). In fact, the chemical, mechanical, thermal and electrical properties of the different allotrope forms are directly correlated to their structure and hybridization state, opening up the possibility to use the same material for a wide range of applications [19]. Herein, the synthesis and application of fullerenes, CNTs and graphene will be discussed for energy generation and storage.

#### Fullerenes

**Figure 4:**
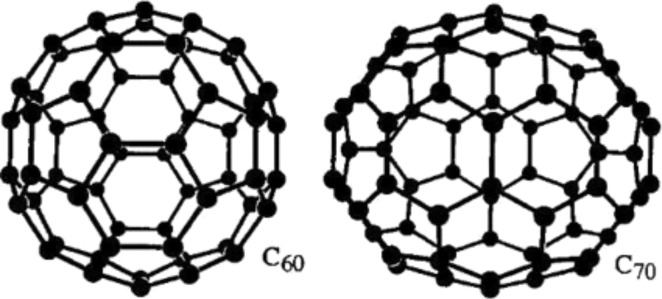
Structure of the most significant fullerenes, C_60_ and the C_70_. All fullerenes exhibit hexagonal and pentagonal rings of carbon atoms. Reprinted with permission from [26]. Copyright (2005) John Wiley and Sons.

Fullerenes are allotropes of carbon, also called buckyballs because of their spherical structure. Fullerenes were predicted and theoretically studied before their experimental discovery by Japanese [20] and Russian [21] researchers in the 1970s, but it was only in the mid-1980s that H. Kroto, R. Smalley and R. Curl were able to detect the first fullerene molecule obtained by laser vaporization of carbon from a graphite target using mass spectroscopy [22]. The name fullerene (C_60_) was dedicated to the architect Buckminster Fuller who was famous for designing and building geodesic domes [23]. The C_60_ molecule is composed of hexagonal and pentagonal faces to form a spherical structure similar to a soccer ball with a diameter of ≈10 Å. This icosahedral symmetry was only first experimentally demonstrated in the 1990s by nuclear magnetic resonance [24]. The C_60_ was the first 0D allotrope of carbon discovered but it is not the only one. In fact, large quantities of C_70_, C_76_, C_78_, C_84_ and even larger clusters, such as C_240_ and C_330,_ have also been synthetized and studied [25]. In particular, C_70_ can be seen as a C_60_ molecule with a belt of five hexagons around the equatorial plane and exhibits a more oval shape (Figure 4) [26].

The main properties of C_60_ are [25]:

- Young’s modulus, ≈14 GPa
- Electrical resistivity, ≈1014 Ω m
- Thermal conductivity, ≈0.4 W/mK
- Band gap, 1.7 eV

The other fullerene species show similar properties to C_60_. Depending on the application, different fullerenes are chosen according to their slight differences in properties.

**Figure 5:**
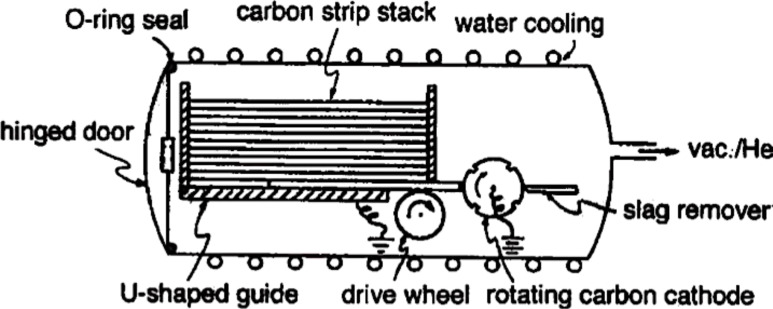
Schematic depiction of an auto-loading version of an arc-discharge apparatus used for fullerene production. Reprinted with permission from [29]. Copyright (1969) Royal Society of Chemistry.

Historically, the first technique to synthesize fullerenes was based on laser ablation of graphite targets in a He gas; however, this does not produce large quantities of the material and is thus mostly used for research studies. The common method to produce large quantities (several grams per day) of fullerenes was first developed by Kratschmer et al. [27] and consisted of an AC arc discharge between high purity graphite rods in 100–200 Torr of helium (He) or Argon (Ar) gas. The temperature required for fullerene formation is about 2000 °C. At this temperature, the electrodes evaporate carbon, forming soot that contains fullerenes, which then condenses on the cool walls of a reactor made of stainless steel or Pyrex [28]. Typically the concentration of fullerenes found in the soot is about 15% (≈13% C_60_ and ≈2% C_70_). Several setups have been proposed but the one proposed by Bezmelnitsyn et al. [29] is one of the most popular because of the large quantity of material produced. It uses 24 carbon strip, auto-loaded anodes that are subsequently consumed during the process. The cathode consists of a rotating carbon wheel, which passes over a scraper to remove the accumulated carbon powder (Figure 5). This method leads to a fullerene production of about 100–200 g/day.

**Figure 6:**
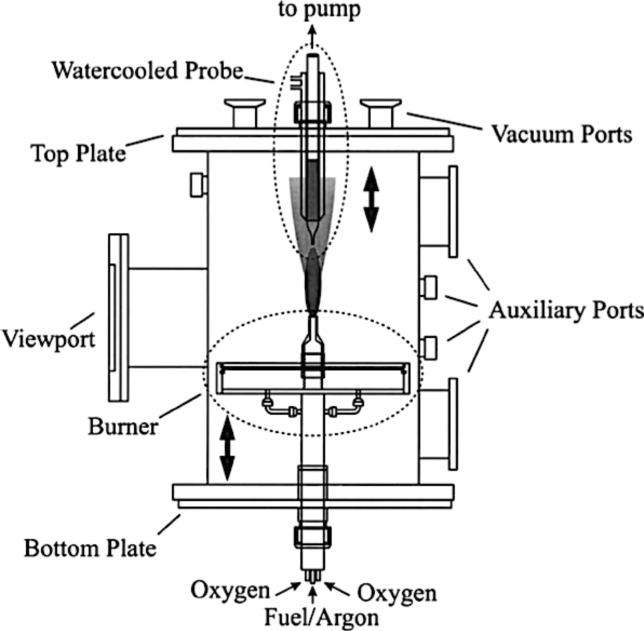
Diffusion flame chamber for fullerene production. Reprinted with permission from [31]. Copyright (2000) Elsevier Limited.

Another method based on the combustion of benzene in an oxygen deficient environment has been proposed for the formation of C_60_ and C_70_ [30–31]. Benzene diluted with Ar is injected along the central axis of a combustion chamber and oxygen is fed at 12–40 Torr through a large diameter porous plate. The flame chamber is usually composed of a burner and a fuel injection system mounted in the bottom of the chamber. The chamber has viewing ports to monitor the process and to insert the plate where the final material is deposited (Figure 6) [31].

**Figure 7:**
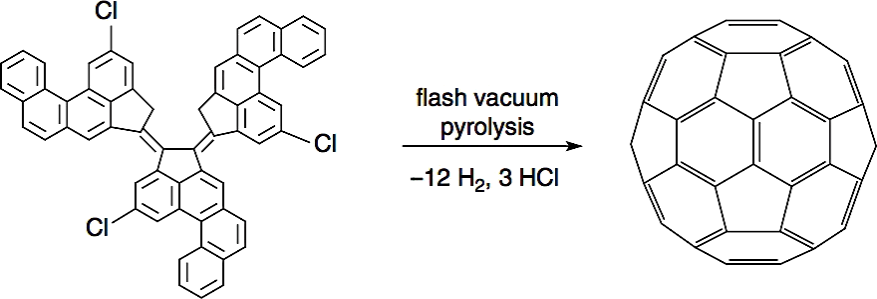
Formation of C_60_ through dehydrogenation/dehydrochlorination. Reprinted with permission from [32]. Copyright (2002) The American Association for the Advancement of Science.

Chemical methods have also been proposed to synthetize fullerenes, but the production yield is so low that it is considered only for research purposes. For example, C_60_ can be produced by the pyrolysis hydrogenation of naphthalene, corannulene or others, but it requires high energy. Dehydrohalogenation of precursors can also be a valid chemical method to form C_60_ from a chloroaromatic compound, as shown for example in Figure 7 [32].

Fullerenes can also be modified by putting dopants inside the structure. Fullerenes with atoms enclosed inside are called “endohedral fullerenes”. The endohedral fullerenes are divided into two categories: the endohedral metallofullerenes, where metal atoms (typically transitional metal atoms) are inserted into the structure during the synthesis [33]; and the endohedral nonmetal-doped fullerenes with noble gas such as helium, neon, argon and xenon inserted into the structure [34].

**Figure 8:**
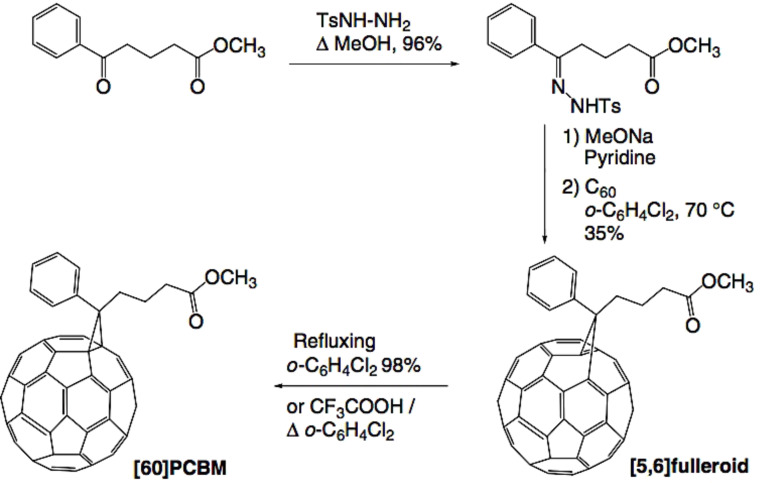
Synthesis of PC_61_BM by reaction between C_60_ and diazoalacane with subsequent refluxing with *o*-dichlorobenzene solution or with trifluoroacetic acid. Reprinted with permission from [26]. Copyright (2005) John Wiley and Sons.

Another fullerene species is the exohedral fullerene or fullerene derivative, which are molecules created by bonding a fullerene with other chemical groups. A typical example of a fullerene derivative is the [6,6]-phenyl-C61-butyric acid methyl ester (PC_61_BM), which is largely used in organic solar cells. Hummelen et al. [35] were the first group to synthesize PC_61_BM by reacting diazoalcane with a C_60_ to reach the [5,6]fulleroid ester and subsequent isomerization to the [6,6]methanofullerene by refluxing it with *o*-dichlorobenzene solution or with trifluoroacetic acid (Figure 8) [36].

#### Carbon nanotubes

Carbon nanotubes (CNTs), discovered by Ijima in 1991 [37], are another allotrope form of carbon with a cylindrical structure. The unique structure of CNTs results in many extraordinary properties. Since the discovery of CNTs, scientists have made great progress in the experimental and theoretical study of their mechanical, electrical and thermal properties. CNTs exhibit remarkable properties including:

- Tensile strength of at least 37 GPa and strain to failure of at least 6% [38–39]
- Young’s modulus, ≈0.62–1.25 TPa [40]
- Electrical resistivity, ≈1 μΩ cm [41]
- Thermal conductivity, ≈3000 W/mK [42]

In addition to their extraordinary properties, the density of CNTs is around 1.33–1.4 g/cm^3^ [40], which is half of the density of aluminium (2.7 g/cm^3^), making them very attractive for lightweight applications.

**Figure 9:**
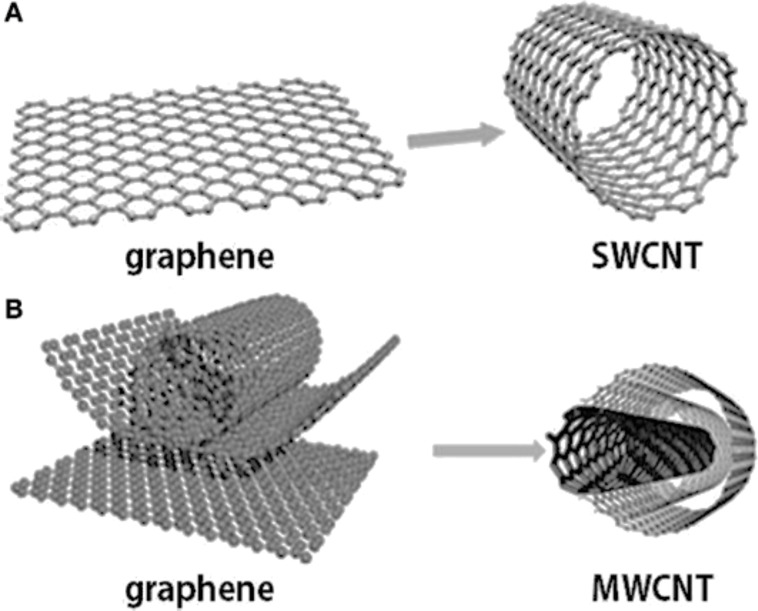
Graphene and carbon nanotubes as a (a) single-walled carbon nanotube (SWCNT) and (b) multiwall carbon nanotube (MWCNT) structure [44].

CNTs are categorized as single-walled carbon nanotubes (SWNTs) and multiwall carbon nanotubes (MWNTs). SWNTs are single graphene sheets rolled up to form a tube, while the MWNTs consist of multiple rolled layers (concentric tubes) of graphene (Figure 9) [43].

**Figure 10:**
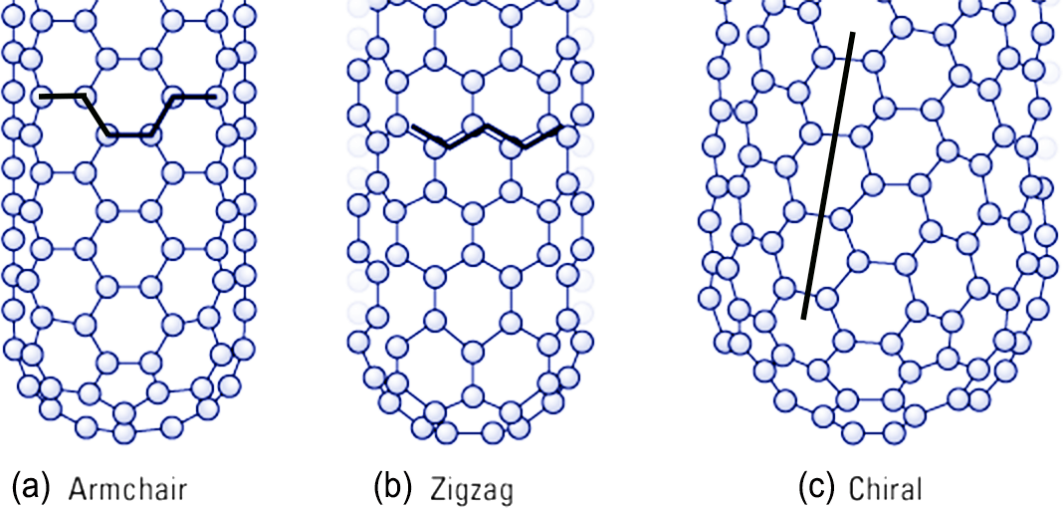
Schematic models for SWCNTs with the nanotube axis normal to the chiral vector, which, in turn, is along: (a) the direction 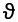 = 30° in an armchair (*n*, *n*) nanotube, (b) the direction 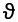 = 0° in a zigzag (*n*, 0) nanotube, and (c) a general 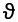-direction in a chiral (*n*, *m*) nanotube.

The manner in which the graphene wraps is indicated by a chiral vector whose components along the base vectors are defined by indices (*n*, *m*). If *m* = 0 the SWNTs are called zigzag; if *n* = *m*, they are armchair, otherwise, they are chiral if *n* ≠ *m* (see Figure 10) [45].

The chiral vector, identified by the index (*n*, *m*), is very important because it strongly affects the electronic properties of the SWNTs. For a given (*n*, *m*) nanotube, if *n* = *m*, the nanotube is metallic; in all other cases, the nanotube is semiconducting with the remarkable situation that when (*n* − *m*) is a multiple of 3, the nanotube has a very small band gap [45]. It was also observed that the energy gap scales inversely with the tube diameter [45].

An important feature of the CNTs is that they have a high aspect ratio (*A* = 10^10^), with *A* = *l*/*d*, where *l* is the length of the CNT that varies from 20 nm to 2 mm and *d* is the diameter of the CNT (typically 0.3–2 nm) [46].

**Figure 11:**
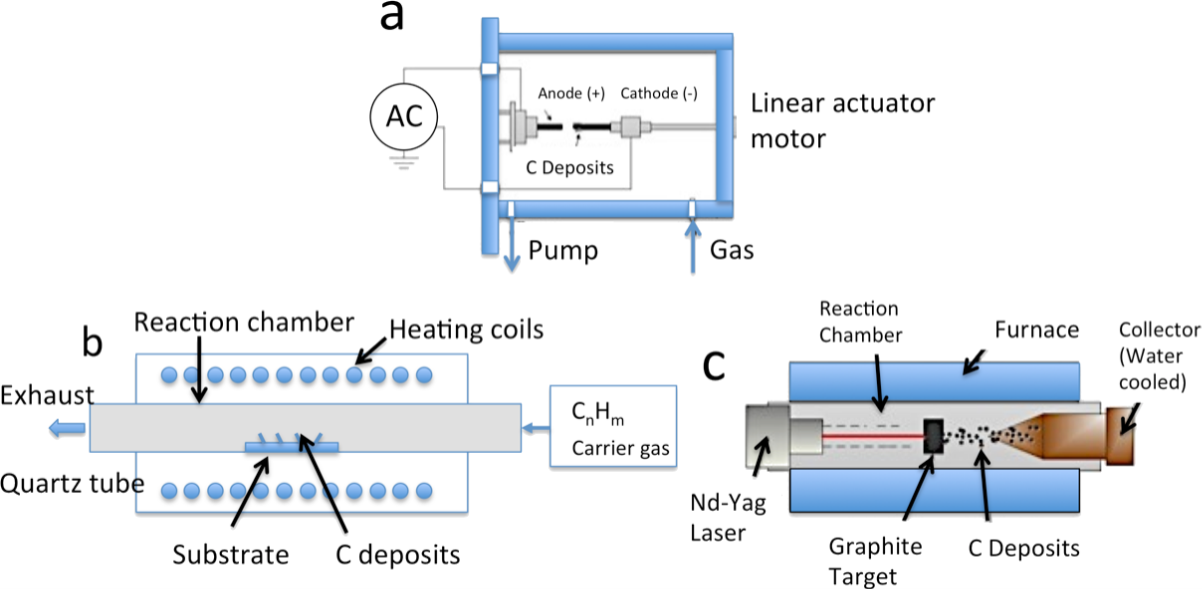
Schematic representation of methods used for carbon nanotube synthesis: (a) arc discharge; (b) chemical vapor deposition; and (c) laser ablation.

There are three main methods to synthesize CNTs, each of which have advantages and disadvantages in terms of quality and length of the nanotubes produced, are [47] (Figure 11):

- Arc discharge: higher batch yield (≈1 g/day) as compared to CVD,
- Laser vaporization: higher batch yield (≈1–10 g/day) as compared to CVD, and
- Chemical vapor deposition (CVD): high quality, most common method with low batch yield (≈30 mg/day).

In the arc-discharge method, the carbon is evaporated by helium plasma ignited by high current passed through an opposing carbon anode and cathode. This method requires the use of a metal catalyst such as cobalt [48]. The nanotubes are typically bound together by strong van der Waals interactions and form tight bundles.

The second method, laser ablation, uses continuous wave [49] or pulsed [50] lasers to ablate a carbon target in a 1200 °C tube furnace. A laser beam evaporates a graphite sample containing 1% nickel and cobalt catalyst particles [51]. In the resulting vapor, the metal aggregates into carbon-saturated catalyst nanoparticles, which instigate the growth of CNTs [48]. These catalyst particles are necessary to produce SWNTs rather than MWNTs [52]. The relative amount of SWNTs, MWNTs, and impurities produced by these methods is dependent on the exact reactor conditions. Impurities include fullerenes, metal catalyst particles encapsulated by graphitic polyhedrons, and amorphous carbon. The majority of impurities can be removed by purification processes based on nitric acid [53]. In both the arc discharge and laser ablation methods, bundles of MWNTs and SWNTs held together by van der Waals forces are generated by the condensation of carbon atoms generated from the evaporation of solid carbon sources.

The third method, chemical vapor deposition (CVD), involves the flow of a hydrocarbon gas over a catalyst in a tube furnace. The catalyst is typically transition metal nanoparticles on a support such as alumina. Materials grown on the catalyst are collected after cooling the furnace to room temperature. The key process parameters are the hydrocarbon and catalyst types, as well as the operating temperature [54]. The production of MWNTs typically involves ethylene or acetylene feedstock with an iron, nickel or cobalt catalyst and operating temperatures of 550–750 °C. SWNTs are produced by using methane or ethane feedstock, similar catalysts, and operating temperatures of 850–1000 °C [55]. Other CVD derivative methods are used that produce CNTs at a reduced growth temperature and increased batch yield. These methods are: plasma-enhanced CVD, where a gas such as C_2_H_2_, CH_4_, C_2_H_4_, C_2_H_6_, or CO is supplied to the chamber and a discharge at high frequency is applied in the chamber [56]; laser-assisted thermal CVD, where a continuous wave CO_2_ laser with medium power is applied perpendicular to a substrate, then pyrolyses sensitized mixtures of acetylene and Fe(CO)_5_ vapor in a flow reactor [57]; and high-pressure catalytic decomposition of carbon monoxide (HiPco), where carbon monoxide and catalyst particles generated from the decomposition of Fe(CO)_5_ flow into a high pressure reactor (up to 10 atm) at temperatures from 800–1200 °C [58]. With the HiPco method, a large yield (>10 g/day) and narrow nanotubes can be produced [59].

#### Graphene

Despite their excellent electrical, mechanical and thermal properties, CNTs are not the only carbon nanomaterial that could play a major role in replacing conventional materials for energy generation and storage devices. In particular, the discovery of the electrical properties of graphene, another allotrope carbon, by Geim, Novoselov and co-workers [60] opened up the potential of this interesting material to being employed in real-world applications. In fact, graphene shows similar or even better mechanical, thermal and electrical properties than CNTs. Moreover, from an engineering point of view, the production and the usage of graphene could be easier when compared to CNTs. Fewer manufacturing parameters, such as chirality and the nature of the nanotubes (SWNTs vs MWNTs), need to be considered and a larger availability of synthesis processes makes it very attractive in the field of material science.

**Figure 12:**
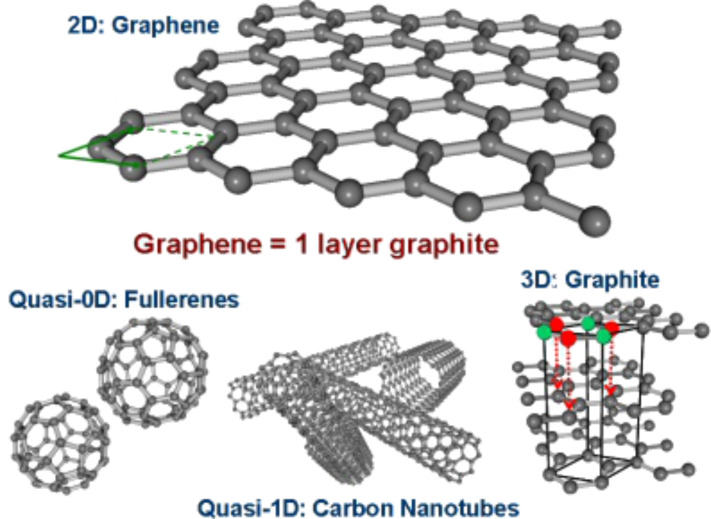
Honeycomb lattice of graphene. Graphene layers can be stacked into graphite or rolled up into carbon nanotubes. The formation of fullerenes requires the incorporation of five-membered rings. Reprinted with permission from [61]. Copyright (2008) John Wiley and Sons.

Graphene is the building block of other important allotropes because it can be stacked to form 3D structures (graphite), rolled to form 1D structures (nanotubes) and wrapped to form 0D structures (fullerenes) (Figure 12) [61]. It consists of a single atomic layer of carbon atoms bonded together in a honeycomb lattice formed by two sublattices, A and B. A single graphene layer has a thickness of 0.34 nm and a carbon–carbon distance of 0.142 nm [60].

The experimentally measured graphene properties are very close to the theoretically predicted limits: high intrinsic mobility at room temperature (250,000 cm^2^ V^−1^ s^−1^) [62–63], high Young’s modulus (≈1 TPa) with an intrinsic strength of 130 GPa [64–65], high thermal conductivity (over 3000 W m^−1^ K^−1^) [66] and excellent optical transmittance (≈97.7%) [67]. Additional graphene characteristics include: high theoretical specific surface area (2630 m^2^ g^−1^) [68], impermeability to gases [69], capability to carry high current densities (a million times higher than copper) [70], anomalous quantum Hall effect (QHE) that appears larger than in other materials [71–72], and zero band gap semiconducting properties with one type of electrons and one type of holes [73] that can also be tuned for different electronic applications [74–75].

**Figure 13:**
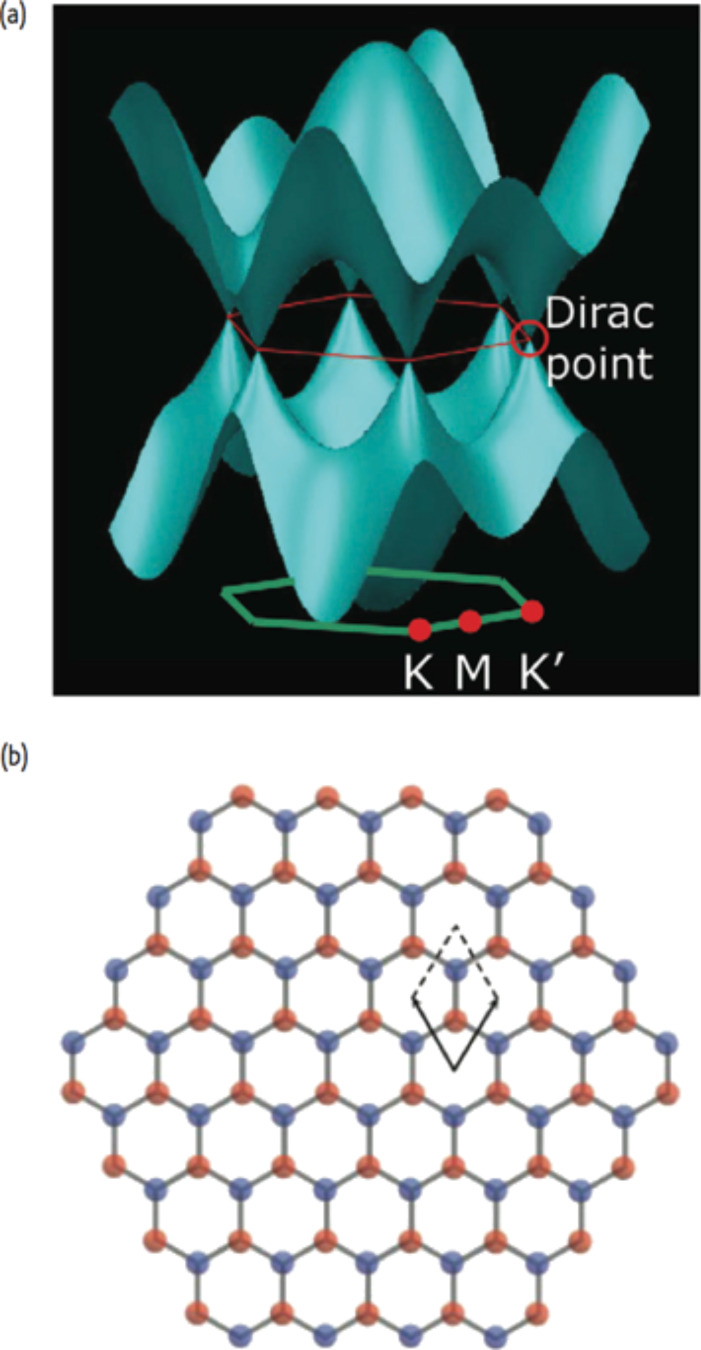
(a) Representation of the electronic band structure and Brillouin zone of graphene; (b) The two graphene sublattices (red and blue) and the unit cell [77].

Most of these extraordinary properties, in particular the electrical and electronic ones, are attributed to the unique band structure that this material has, which was first calculated in 1947 by P. R. Wallace [76]. The valence band, formed by bonding π states and the conduction band, formed by the antibonding π^*^ states, are orthogonal and they touch only at six points (Dirac points indicated as K and K’). The graphene electron dispersion is linear but also very sensitive to external perturbations that can interact with the π-electron of the system (Figure 13) [77].

It should be mentioned that graphene is not the only 2D material today that offers great performance for a wide range of applications [78]. Boron nitride and molybdenum disulfide are examples of other 2D materials that offer the possibility to tune material and device characteristics for a specific application and can even be used in combination with graphene [79–80].

**Figure 14:**
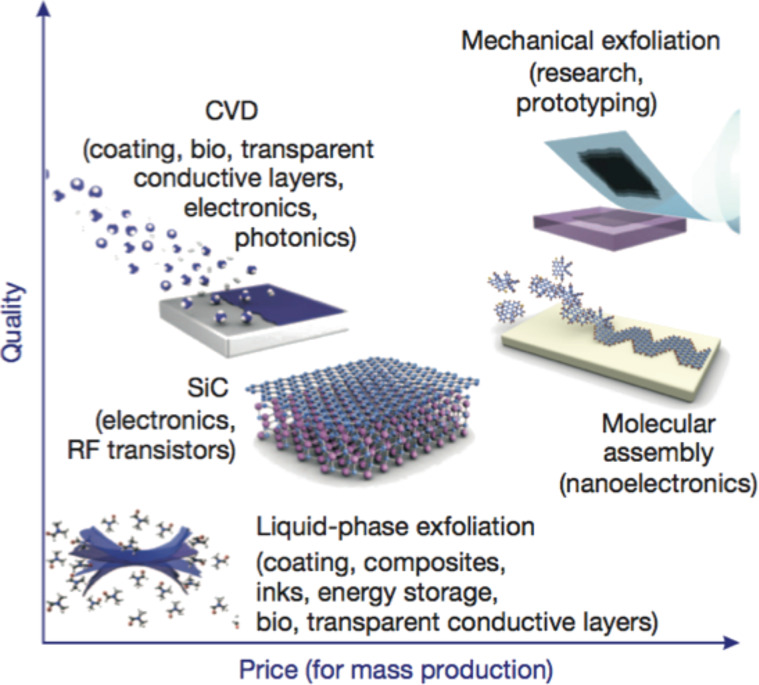
Several methods for the mass production of graphene that allow a wide choice in terms of size, quality and price for any particular application. Reprinted with permission from [81]. Copyright (2012) Nature Publishing Group.

As mentioned previously, the synthesis processes available today to produce graphene can potentially achieve a better quality material with higher batch yield than CNTs, resulting in increased interest from industry in the development of this technology. There are more than ten processes available to synthesize graphene but only the following five can be reasonably considered in terms of quality and material scalability (Figure 14) [81]:

- Mechanical exfoliation
- Chemical exfoliation
- Chemical exfoliation via graphene oxide
- CVD
- Synthesis on SiC

Each of these methods has its advantages and disadvantages in terms of quality, yield production and applications, as summarized in Table 1. In particular, mechanical exfoliation most likely produces the best samples in terms of charge carrier mobility but is probably the worst in terms of scalability. In contrast, CVD methods can produce pristine graphene sheets but only in limited quantities. Large quantities of graphene sheets have recently been obtained with CVD methods using catalytic metal substrates [82–83]. However, associated problems such as harsh conditions (high temperature and high vacuum), complexity of the process, difficulties in transferring to different substrates and high costs still must be resolved. Also, graphene flakes have irregular shapes that require a substrate to control the orientation for high-tech applications [77].

**Table 1:** Properties of graphene obtained by different methods (from [81]).

Method	Crystallite size (µm)	Sample size (mm)	Charge carrier mobility (ambient temperature) (cm^2^ V^−1^ s^−1^)	Applications

Mechanical exfoliation	>1,000	>1	>2 × 10^5^ and >10^6^ (at low temperature)	Research
Chemical exfoliation	≤0.1	Infinite as a layer of overlapping flakes	100 (for a layer of overlapping flakes)	Coatings, paint/ink, composites, transparent conductive layers, energy storage, bioapplications
Chemical exfoliation via graphene oxide	≈100	Infinite as a layer of overlapping flakes	1 (for a layer of overlapping flakes)	Coatings, paint/ink, composites, transparent conductive layers, energy storage, bioapplications
CVD	1,000	≈1,000	10,000	Photonics, nanoelectronics, transparent conductive layers, sensors, bioapplications
SiC	50	100	10,000	High-efficiency transistors and other electronic devices

Other methods based on the chemical exfoliation of graphite and thermal or chemical reduction of graphene oxide can produce graphene on an industrial scale but unfortunately with structural defects that can affect the electronic and electrical properties [84–85].

**Figure 15:**
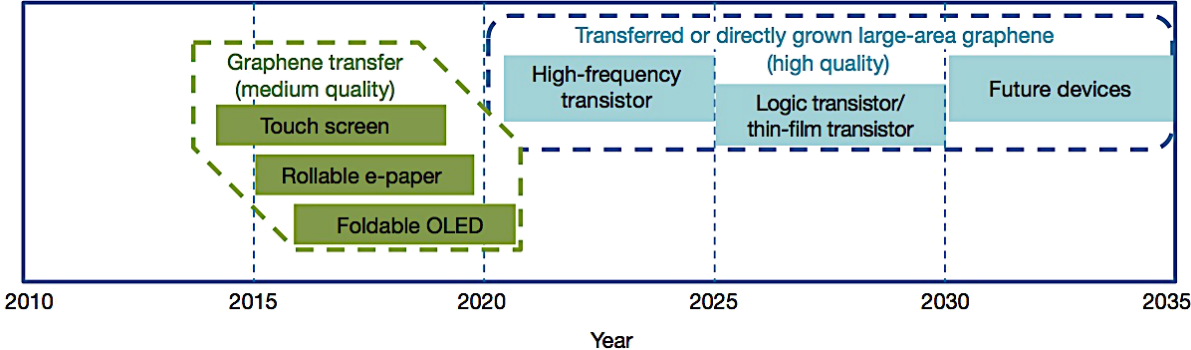
Graphene-based display and electronic devices. Display applications are shown in green; electronic applications are shown in blue. Possible application timeline, based on projections of products requiring advanced materials such as graphene. Reprinted with permission from [81]. Copyright (2012) Nature Publishing Group.

These are the main problems that impede the production of high quality graphene on a large scale. However, a possible application timeline has already appeared in the literature indicating when possible electronic device prototypes could be expected in the future (Figure 15) [81].

**Mechanical exfoliation.** The mechanical exfoliation method was historically the first to be adopted by Geim, Novoselov and co-workers to isolate single layers of graphene [86]. With this method, bulk graphite can be exfoliated into individual graphene sheets by using Scotch tape and then transferred by pressing it onto a substrate, such as Si, SiO_2_ or Ni [87]. Typically, highly ordered pyrolytic graphite (HOPG) is chosen in order to guarantee a product of high quality graphene crystallites. The main advantages of this method are the ability to complete this process at room temperature and with inexpensive equipment. However, in terms of scalability, it performs the worst and can thus only be considered for research purposes.

**Chemical exfoliation.** It is well known that the van der Waals forces that bond together the graphene sheets to form graphite are particularly weak and can be broken by external mechanical force. A common example of this can be seen with the usage of pencils or solid lubricants based on graphite.

Graphene can also be exfoliated from graphite by chemical methods, the process of which is very similar to the dispersion of polymers in particular solvents. This method can be explained by enthalpy and charge transfer between the graphene layers and the solvent molecules [88–89]. In particular, it was discovered that effective solvents are those with a surface energy similar to graphene (≈0–50 mJ m^−2^) [88]:

[1]
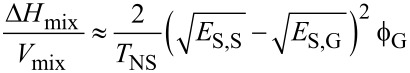


where Δ*H*_mix_ is the enthalpy of the mixing, *V*_mix_ is the volume of the mixture, *T*_NS_ is the thickness of a graphene nanosheet, *E*_S,S_ and *E*_S,G_ are the surface energies of the solvent and graphene, and 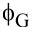 is the volume fraction of graphene dispersed in the solution. From Equation 1, it is clear that the enthalpy of the mixing is minimal when the two surface energies are close or equivalent meaning that the exfoliation will take place with mild sonication [88,90]. Furthermore, some solvents are more suitable because they match the graphene surface energy, such as *N*,*N*-dimethylformamide (DMF), benzyl benzoate, γ-butyrolactone (GBL), 1-methyl-2-pyrrolidinone (NMP), *N*-vinyl-2-pyrrolidone (NVP) and *N*,*N*-dimethylacetamide (DMA), while ethanol, acetone and water are considered poor solvents for exfoliation [91–92].

Other solvents, such as ionic liquids [93] and chlorosulfonic acid [94], have also been proposed for exfoliating graphite but the exfoliation mechanism has been explained differently. In fact, it has been demonstrated in these cases that there is a charge transfer between the solvent and the graphite layers allowing the exfoliation to take place. Therefore, the graphene sheets could be positively or negatively charged with varying donor and acceptor numbers depending on the solvents.

Surfactants and polymers can also contribute to graphite exfoliation if mixed with particular solvents such as water. Specifically, they can change the wettability and prevent aggregation due to electrostatic repulsion [95].

The main problem with the liquid-phase exfoliation method is that it produces graphene for films that is not completely transparent (from 80% to 90%) with high sheet resistance (from 8 to 5 kΩ) [88,90]. The increased sheet resistance is due to damage caused by the sonication during exfoliation.

**Figure 16:**
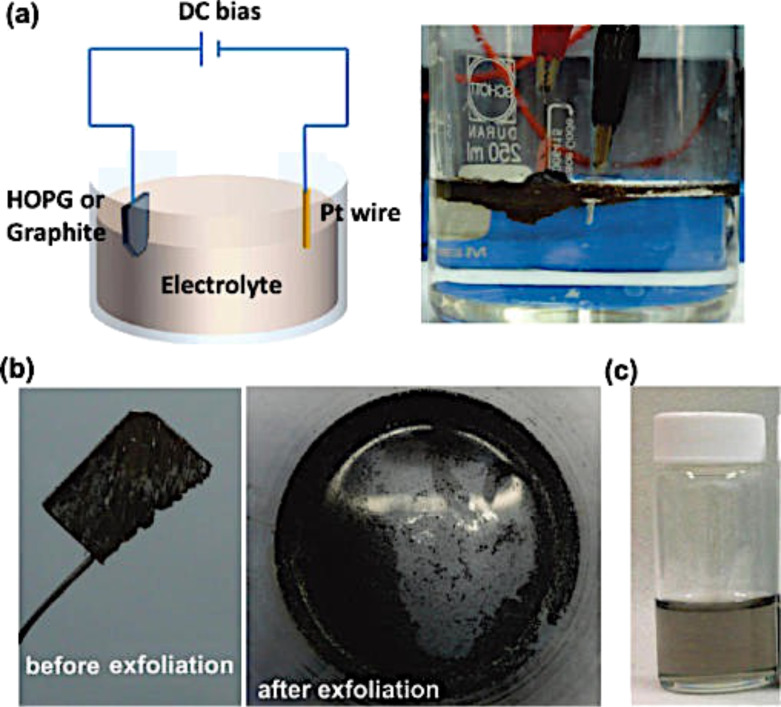
(a) Schematic illustration and photo of the electrochemical exfoliation process for graphite. (b) Photos of the graphite flakes before and after electrochemical exfoliation. (c) Photo of the dispersed graphene sheets in a DMF solution. Reprinted with permission from [96]. Copyright (2011) American Chemical Society.

For these reasons, electrochemical exfoliation methods have recently been employed to produce better quality graphene with a faster process [96–97]. In the electrochemical exfoliation method, the graphite or HOPG is usually connected to an electrode (anode). The counter electrode (cathode) is usually a platinum (Pt) wire and the setup is usually placed in an acidic solution (Figure 16). The complete exfoliation takes place in 15–30 min with voltages varying from 4–10 V. The final graphene flakes produced are usually employed to make a thin film transparent electrode with a sheet resistance of 210 Ω/sq with 96% transparency [96] or to make thin film supercapacitors with capacitance values of over 1 mF/cm^2^ [97].

**Chemical or thermal reduction of graphene oxide.** Graphene oxide (GO) is a semiconducting material originating from graphene research and can be considered a precursor of the graphene synthesis by chemical or thermal reduction [84–85,98–99]. It has recently attracted significant interest because of its potential as a precursor material for high yield production and functionalization of graphene sheets.

**Figure 17:**
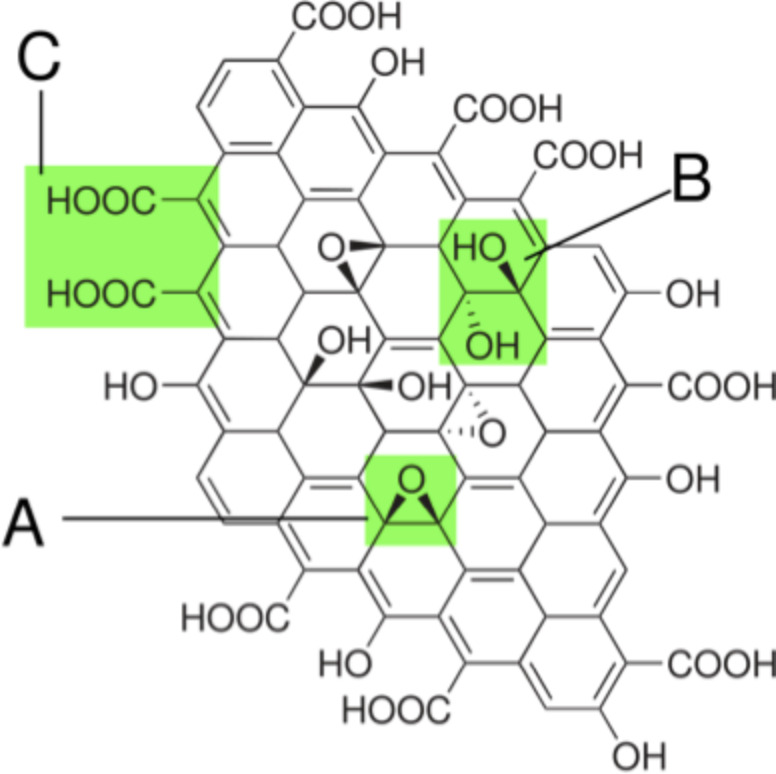
Chemical structure of graphene oxide with functional groups. A: Epoxy bridges, B: hydroxy groups, C: pairwise carboxy groups (image from https://en.wikipedia.org/wiki/Graphite_oxide, structure from [102]).

The main difference between GO and graphene is that GO consists of epoxy (C–O–C) trigonally bonded in sp^2^/partially sp^3^ configurations, hydroxyl groups (C=O) in sp^3^ configuration displaced above or below the graphene plane, and of some carboxylic groups (–COOH) at the edges of the graphene plane (Figure 17) [100–102].

GO exhibits excellent mechanical, optical, thermal and electronic properties that are similar to graphene because of its specific 2D structure and the presence of various oxygenated functional groups. From an electronic point of view, the GO, as synthesized, is typically insulating and has a high sheet resistance around 10^12^ Ω/sq [103]. This intrinsic insulating nature is strongly related to the amount of C–O–C and C=O groups in this material. However, chemical and thermal treatments can help to reduce the GO in order to remove the oxygen groups with a resulting increase in the conductivity and a tuning of the intrinsic properties from insulating to semiconducting [104]. As compared with pristine graphene, GO presents enhanced chemical activity because of the presence of a large number of oxygen containing functional groups and structural defects.

GO can be chemically produced from graphite oxide (also obtained from graphite by treatment with strong oxidizers), which is a compound of carbon, oxygen, and hydrogen in variable ratios. The first to synthetize the graphite oxide was Benjamin C. Brodie in 1859 [105] by treating graphite with a mixture of potassium chlorate (KClO_3_) and fuming nitric acid (HNO_3_). Then, in 1957, Hummers and Offeman found a safer, quicker, and more efficient way, which is largely used today in the scientific community and is based on a mixture of sulfuric acid (H_2_SO_4_), sodium nitrate (NaNO_3_), and potassium permanganate (KMnO_4_) [106]. Recently, Hummers-modified methods have been proposed in order to produce a higher fraction of well-oxidized hydrophilic carbon material with a more regular structure where the basal plane of the graphite is less disrupted [107].

**Figure 18:**
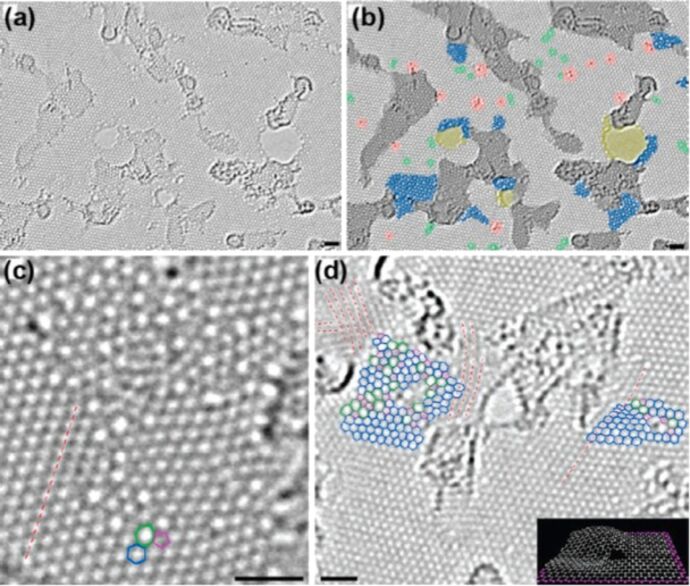
Atomic resolution, aberration-corrected TEM image of a single layer, H-plasma-reduced GO membrane. (a) Original image, (b) original image with false colored to highlight the various features; (c) atomic resolution TEM image of a nonperiodic defect configuration; and (d) partial assignment of the configurations in defect areas, where the inset shows a structural model clearly showing the strong local deformations associated with defects. All scale bars are 1 nm. Reprinted with permission from [111]. Copyright (2010) American Chemical Society.

The attractive property of GO is that it can be thermally and chemically reduced to produce graphene, usually called reduced GO (rGO) [108–109]. The name rGO is given in order to differentiate it from the pristine graphene. In fact, residual, functional groups and defects break the conjugate structure, decreasing the carrier mobility and concentration. Current research in rGO is not only focused on removing the functional groups but also on recovering the network of the graphene lattice [110]. In fact, rGO results in a much lower conductivity when compared to pristine graphene because of large areas of defects as demonstrated by TEM images (Figure 18) [111].

**Figure 19:**
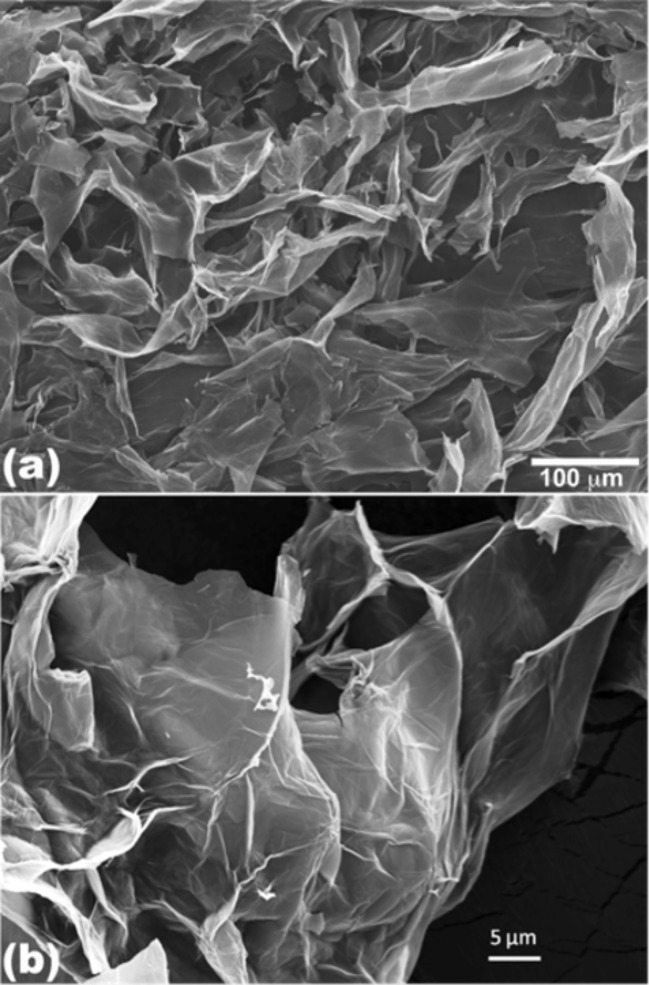
(a) Low magnification and (b) high magnification SEM images of graphite oxide flakes [112].

GO and rGO can be easily distinguished by standard optical observation [109]. rGO usually has an increased charge carrier concentration and mobility that results in improved light reflection when deposited onto a metallic substrate as compared to a GO film deposited on the same substrate. Additionally, when immersed in a solvent such as DMF, the GO solution develops a brown color while rGO appears black. The microscopic character of GO flakes reveals a certain wrinkling of thin and aggregated flakes stacked onto each other. Their lateral size ranges from 100 nm to several μm [112] (Figure 19).

**Figure 20:**
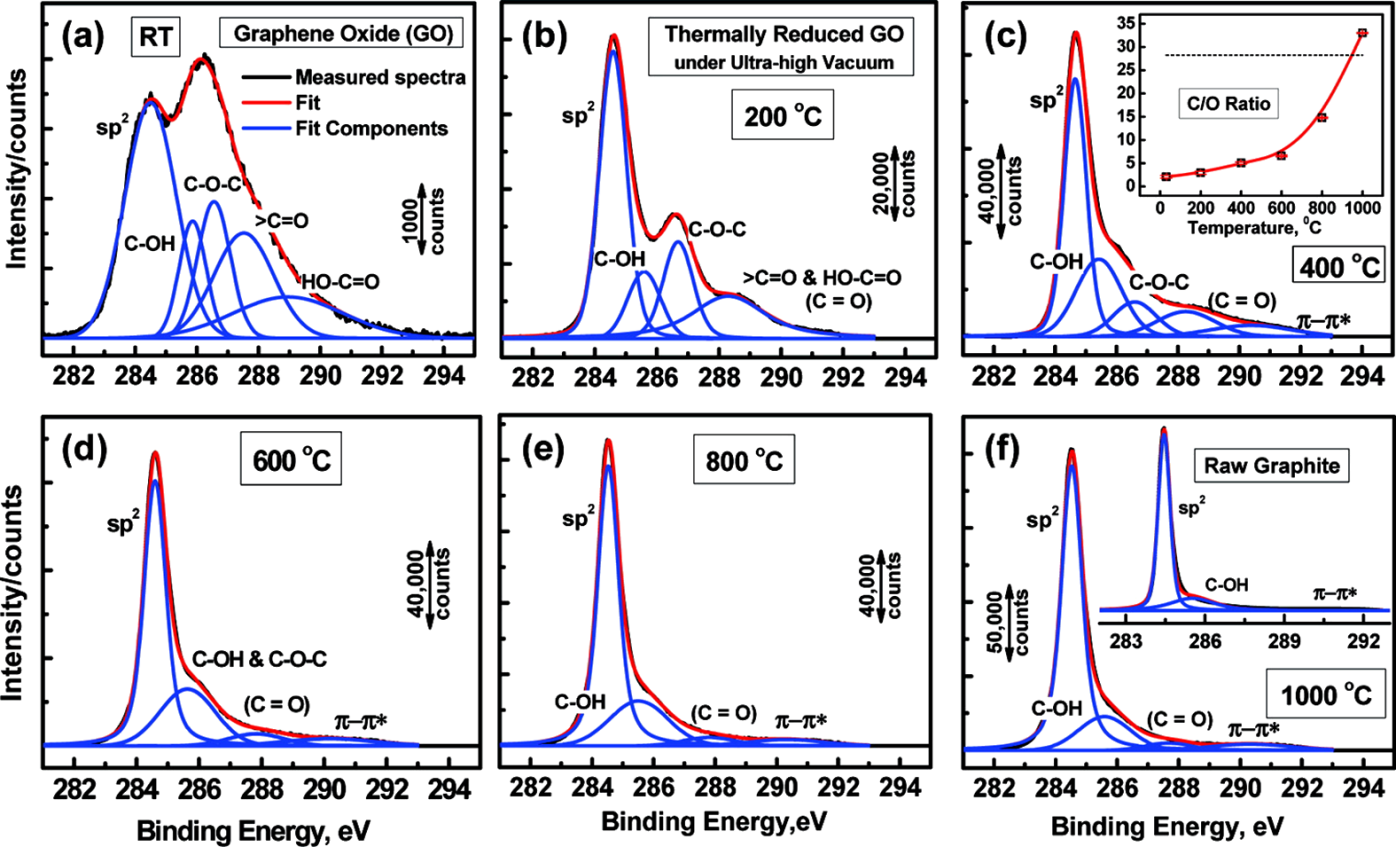
High resolution C 1s XPS spectra: deconvoluted peaks with increasing reduction temperature (*T*_r_). (a) Room temperature; (b) 200 °C; (c) 400 °C, where the insert shows the C/O ratio as a function of *T*; (d) 600 °C; (e) 800 °C; and (f) 1000 °C, where the insert shows the C 1s spectra for the graphite precursor. Reprinted with permission from [116]. Copyright (2011) American Chemical Society.

Before reduction, the C/O ratio is typically 4:1 to 2:1 [113–114] and can be reduced to 12:1 [115] or even to 246:1 [99], as was recently obtained. The C/O ratio is usually characterized by X-ray photoelectron spectroscopy (XPS) because of the possibility to easily identify all the species and their percentage values in the material (Figure 20) [116–117].

**Figure 21:**
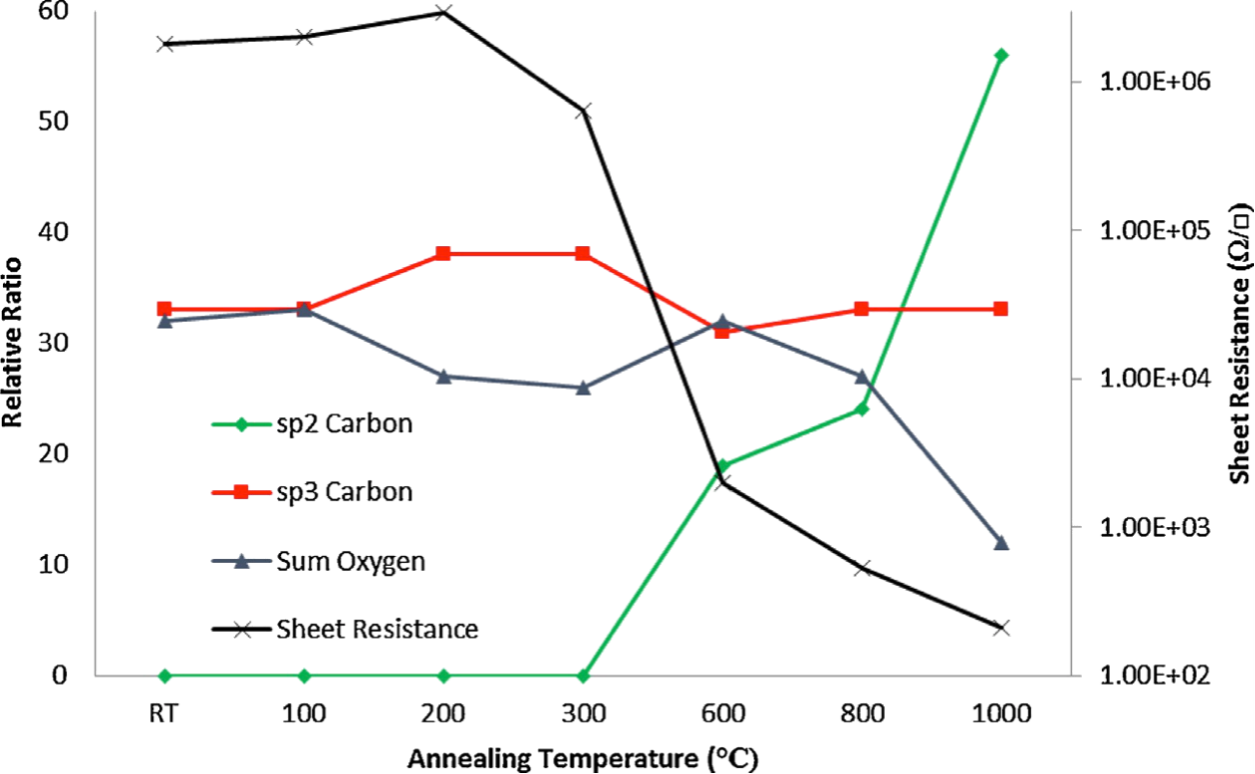
Plot of sheet resistance against annealing temperature with a comparison to key carbon and oxygen ratios. It should be noted that the sheet resistance has been plotted on a logarithmic scale. Reprinted with permission from [119]. Copyright (2015) Elsevier.

The thermal reduction of GO is usually carried out by annealing films or powders in the presence of inert or reducing gases or in vacuum. The annealing temperature certainly affects the properties of the rGO produced. In particular, it was found that the C/O ratio could increase from more than 7 to 13 if the temperature was increased from 500 to 750 °C [118]. The ratio of C/O is also directly connected to the conductivity. In fact, Pei and Cheng [109] demonstrated that the conductivity increased from 50 to 550 S/cm for annealing temperatures of 500 and 1100 °C, respectively. This result has been recently confirmed and explained in detail by Chambers et al. [119] and was found to be related to the loss of oxygen (Figure 21).

Wu et al. [120] used an arc discharge system (instead of a typical furnace) to exfoliate and reduce graphite. With this method, they were able to obtain graphene sheets with a conductivity of 2000 S/cm and a C/O ratio of ≈18 due to the arc discharge system that reached temperatures of over 2000 °C for a short time.

Not only the temperature but also the annealing atmosphere is very important to determine the quality of the resulting rGO. Becerril et al. [103] demonstrated that at 1000 °C, the quality of the vacuum was critical for high quality rGO because of the reaction with residual O_2_ molecules. For this reason, the usage of reducing gases such as H_2_ can help to decrease the required annealing temperature to 450 °C and still give a high C/O ratio of ≈15, as was demonstrate by Wu et al. [120]. Li et al. [121] instead used a mixture of ammonia and argon (2 Torr, NH_3_/Ar (10% NH_3_), 500 °C) to produce good quality, doped rGO.

The drawbacks of thermal reduction are the high energy consumption due to high temperature and time consumption, given that the GO must slowly reach higher temperatures in order to prevent explosion of the material. For these reasons, other heating approaches based on microwave irradiation [122] and photoirradiation have been considered due to the simplicity of these systems and the reduced exposure time [123].

A cheaper and easy way to reduce GO is by chemical reduction, which is usually done at room temperature or with low heating [109]. Among the many chemical reagents that could reduce GO, hydrazine and its derivatives are probably the most widely used by simple addition to an aqueous GO solution [124–127]. The C/O ratio can reach values above 10 and a conductivity of 99.6 S/cm [84,128]. Other compounds based on metal hybrids such as sodium hydride and sodium borohydride have also been used to specifically remove the C=O species. The main problem that occurred is that sodium borohydride, for example, does not reduce the epoxy and carboxylic groups well [129], and for this reason, it should be utilized after treating the GO with concentrated sulfuric acid at 180 °C. With this method, Gao et al. [99] were able to obtain a C/O ratio of 8.6 and a conductivity of about 16.6 S/cm. Unfortunately, these values are still low when compared to the rGO obtained from the hydrazine derivative compounds.

Other reducing agents such as ascorbic acid (C_6_H_8_O_6_) and hydroiodic (HI) acid have been recently proposed because of their potential to obtain higher quality rGO as compared with the product obtained from hydrazine derivative compounds. Fernández-Merino et al. [128] were able to obtain rGO with a C/O ratio of 12.5 and a conductivity of 77 S/cm with ascorbic acid while Moon et al. [130] obtained rGO with a C/O ratio of 15 and a conductivity of 300 S/cm with HI acid.

Derivative approaches of the chemical method have also been employed to reduce GO: photocatalyst reduction where the GO mixed with TiO_2_ particles is exposed to ultraviolet (UV) irradiation [131]; electrochemical reduction with an inert electrode placed in an aqueous buffer solution containing GO where cyclic voltammetry scans are performed between certain voltage ranges [132]; solvothermal reduction where the sealed vial containing GO in a solvent can withstand high temperature and vapor pressure [133].

Much effort has been made by the research community to make the chemical or thermal reduction processes of GO effective; however, the final product is still lacking in terms of quality when compared to pristine graphene. It should be mentioned that mildly oxidized GO has been recently proposed because it could preserve the conjugated structure with few defects [134].

**Figure 22:**
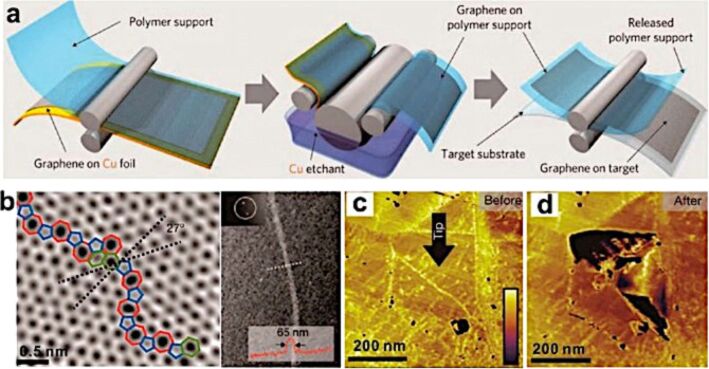
CVD graphene. (a) Schematic of the transfer of graphene produced on Cu using the roll-to-roll method. (b) Two types of graphene boundaries: aperiodic heptagon/pentagon pairs and overlapped bilayer regions. (c,d) Tears along the graphene grain boundaries after indentation. Reprinted with permission from [135]. Copyright (2014) American Chemical Society.

**Chemical vapor deposition (CVD).** The CVD method is commonly used to produce large-area uniform graphene films [77,135]. Similar to the CVD method used to grow CNTs, graphene can be grown from gases containing C on catalytic metal surfaces or by surface segregation of C dissolved in metals such as Fe [136], Ni [137], Co, Pt and Pd [138]. The pioneer who discovered single layer graphite on Pt was S. Hagstrom in 1965 [139], but the first to interpret it as a single layer was J. W. May in 1969 [140]. The CVD and surface segregation methods can also coexist causing the carbon atoms coming from the gas source to diffuse into the metal. The process is very difficult to control, and especially so in polycrystalline metals where the grain boundaries act as nucleation sites for multilayer growth [141]. For this reason, single crystal and atomically smooth metals are usually preferred to grow high quality, monolayer graphene. Also, the choice of the metal, catalytic substrate is very important to avoid the diffusion of the carbon atoms into the metal. For example, Cu surfaces are probably the best choice for a pure CVD process with the formation of a monolayer graphene because the diffusion of C atoms in Cu is very low (0.001 atom % at 1000 °C) [83,142]. The CVD process on Cu foils can be scaled using a roll-to-roll technique, allowing for a 30 inch graphene film for a transparent electrode (Figure 22a) [82]. However, even this method does not guarantee perfect graphene in terms of quality. In fact, the graphene produced is mostly polycrystalline with aperiodic heptagon/pentagon pairs [143] or overlapped bilayer regions [144] at the grain boundaries (Figure 22b). It has also been demonstrated that the presence of grain boundaries can reduce the mechanical and electrical properties of the graphene (Figure 22c,d) [143,145].

**Figure 23:**
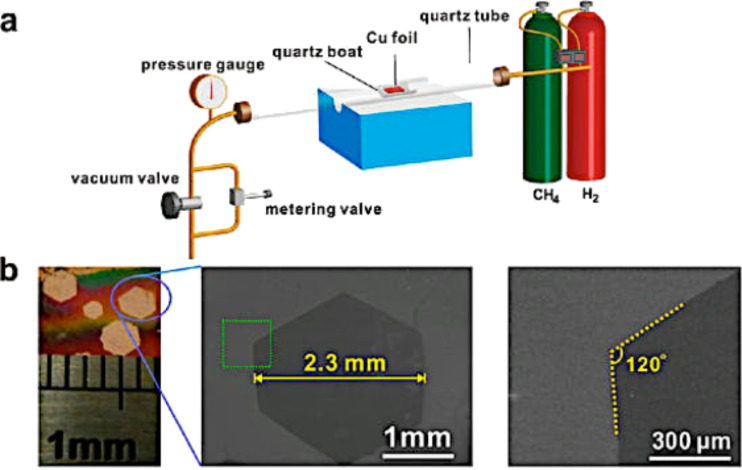
Millimeter-sized graphene grains produced on polished and annealed Cu foils. (a) Schematic of the controlled pressure CVD system. (b) Typical optical and scanning electron microscope (SEM) images of as-produced millimeter-sized graphene grains on pretreated Cu foils. Reprinted with permission from [135]. Copyright (2014) American Chemical Society.

To polish the commercial Cu foil, which is generally covered with a protective layer, the electrochemical method is commonly used, followed by treatment in a CVD system at 2 atm of H_2_ for 7 h to reduce the defects. In this way, an ≈2.3 mm wide monolayer of graphene with mobility of ≈11,000 cm^2^ V^−1^ s^−1^ was synthesized (Figure 23) [146].

**Figure 24:**
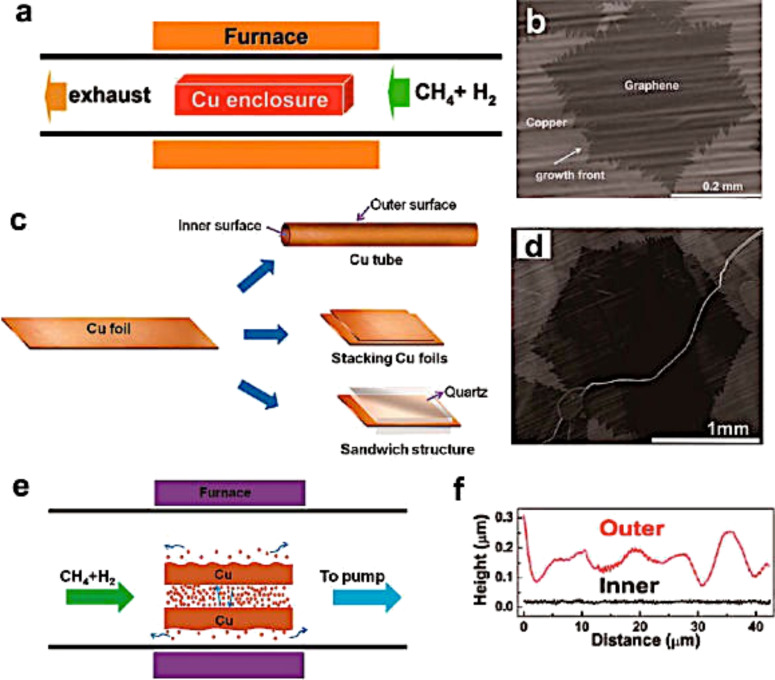
Millimeter-sized graphene grains produced on the inside of enclosure-like Cu structures. (a) Schematic of Cu enclosures for graphene growth. (b) An SEM image of graphene grains grown on the inner surface of a Cu enclosure. (c) Schematic of the Cu tube, stacked Cu foils, and Cu foil between two quartz slides. (d) Typical SEM image of one graphene grain grown on the inner surface of the tube-like Cu structure. (e) Reduction of loss of Cu by evaporation due to redeposition of Cu in a confined space. (f) Height profiles on the inner surface (the black curve) and the outer surface (the red curve) of a tube-like Cu structure after annealing. Reprinted with permission from [135]. Copyright (2014) American Chemical Society.

Enclosure-like Cu structures have also been used by Ruoff’s group to grow large single crystal graphene (≈0.5 mm) [147–148]. Specifically, the Cu was electrochemically polished and then rolled into a tube before being placed in the furnace. They demonstrated that, with this method, the Cu inner surface is smoother than the outer one, allowing the formation of millimeter-sized graphene (Figure 24).

**Figure 25:**
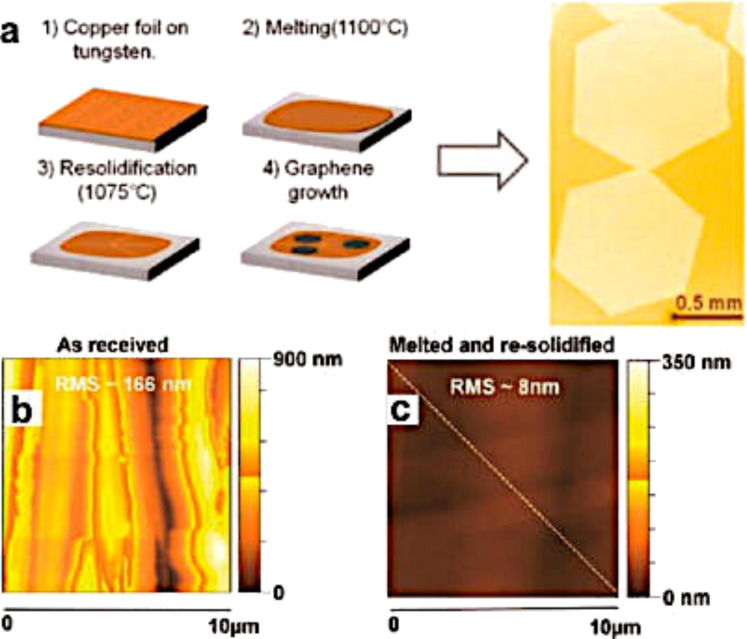
Millimeter-sized graphene grains made on resolidified Cu. (a) Schematic of the Cu resolidification process on a tungsten substrates (left) and an optical image of millimeter-sized hexagonal graphene grains grown on resolidified Cu (right). AFM topographical images of various copper surfaces: (b) as-received and (c) resolidified. Reprinted with permission from [135]. Copyright (2014) American Chemical Society.

Mohsin et al. [149] also showed that the Cu surface morphology is very important for graphene nucleation. In fact, by melting and resolidifying Cu substrates, they were able to obtain a piece of monolayer graphene grains with a size of about 1 mm due to the reduction in the Cu roughness from 166 to 8 nm after treatment (Figure 25).

Another approach, reported by Zhou et al. [150], is to anneal Cu foils in Ar to maintain the catalytically inactive Cu_2_O layer and to extend the graphene growth to 48 h. With this method, they were able to achieve 5 mm wide, monolayer graphene with a high carrier mobility of 16,000 cm^2^ V^−1^ s^−1^. The groups of Luo and Ruoff then adopted a similar strategy and were able to produce centimeter-scale, single crystal graphene [151–152].

The drawbacks of the CVD process are: (1) it is expensive because a large amount of energy is required, (2) the transfer to dielectric (or other) substrates is not easy to achieve, and (3) controlling the crystallographic orientation is critical for many electronic applications. However, the breakthrough that would make this technology viable for large scale production would be to develop a low temperature CVD process (e.g., plasma-enhanced CVD) that could produce large area, high quality graphene on any type of substrate.

**Epitaxial growth on SiC.** Graphene growth on silicon carbide (SiC) has also been extensively explored as it results in wafer-scale growth. Additionally, SiC is an excellent substrate for many electronics applications, avoiding the need to transfer to another substrate. High-quality graphene with a controlled thickness and a specific crystallographic orientation can in fact be grown on commercially available, semiconducting or insulating SiC wafers. For these reasons, this process is very attractive for industrial applications because it can easily be integrated with conventional silicon technology.

SiC is a semiconducting material that can be found in 250 crystalline forms [153]. A large family of similar crystalline forms can be catalogued in particular structures called polytypes, which all present different physical properties.

**Figure 26:**
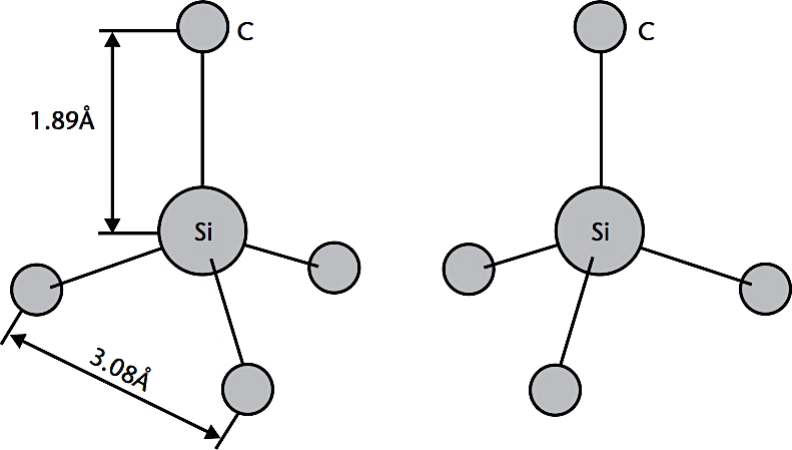
The characteristic tetrahedron building block of all SiC crystals. Four carbon atoms are covalently bonded with a silicon atom in the center or vice versa. Two types exist: one is rotated 180° around the *c*-axis with respect to the other as shown [154].

All the polytypes show a similar local chemical environment for both the carbon and silicon species. Specifically, each C (or Si) atom is situated above the center of a triangle of Si (or C) atoms and underneath a Si (or C) atom belonging to the next layer in a tetrahedral co-ordination. The distance between neighboring silicon or carbon atom is ≈3.08 Å, while the distance between the C atom to each of the Si atoms (Si–C bond length) is ≈1.89 Å. A second type of building block also exists that is identical but rotated 180° with respect to the first (Figure 26) [154]. These units are periodically repeated in closed-packed layers, whose stacking sequence gives rise to the different polytypes.

The two major polytypes are α-SiC and β-SiC. The α-SiC exhibits a hexagonal crystal structure (similar to wurtzite) and is usually formed at temperatures above 1700 °C. β-SiC exhibits a cubic crystal structure with a stacking sequence of ABCABC along the (111) direction, which is typical of a zinc blende crystal structure (similar to diamond) and is formed at temperatures below 1700 °C. The α-SiC and the β-SiC can also be catalogued with the Ramsdell classification scheme where a number indicates the layer and a letter indicates the Bravais lattice type, such as cubic (C), hexagonal (H) or rhombohedral (R) [155].

**Figure 27:**
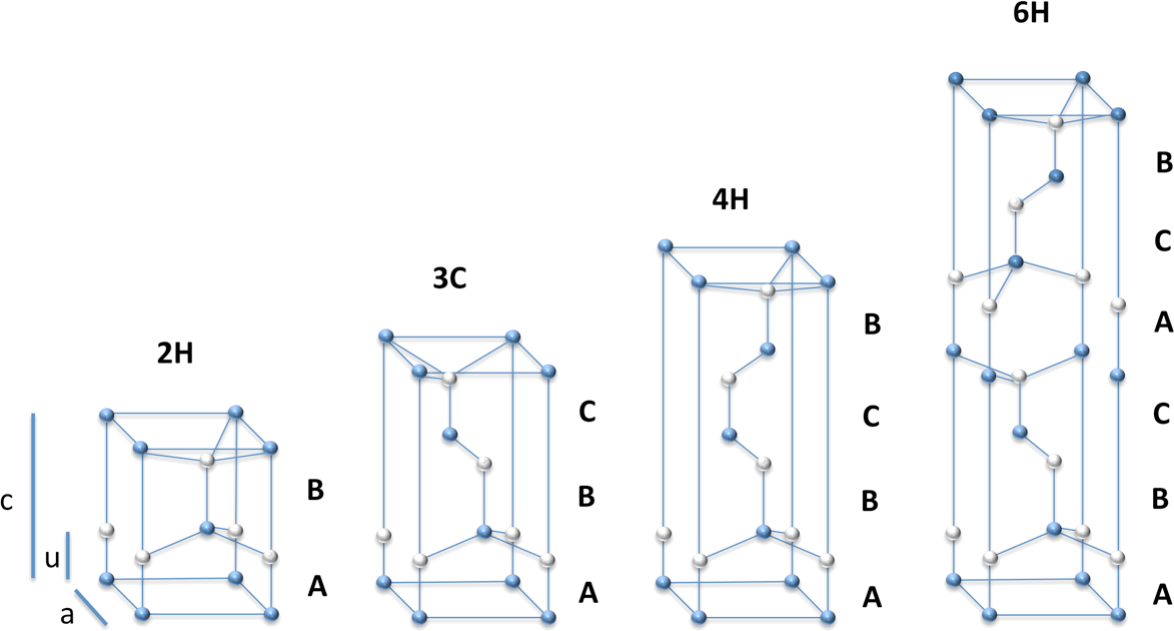
Schematic representation of the stacking sequence of hexagonal SiC bilayers for 2H, 3C, 4H and 6H polytypes.

For example the α-SiC can also be called 2H-, 4H- or 6H-SiC, depending on the unit cell, while β-SiC can also be called 3C-SiC because of the ABC stacking [156] (Figure 27).

The formation process of graphene on SiC is also called graphitization. It consists of the sublimation of Si atoms from the SiC surface caused by high temperatures with a consequent rearrangement of the carbon atoms on the surface to form a graphene lattice [157–159]. Graphene can be grown on both terminated C- or Si-faces, but Si allows for better control over the number of graphene layers and gives a uniform coverage with an azimuthal orientation that is determined by the crystal structure [160].

Hexagonal polytypes of SiC (such as 6H and 4H) with orientation (0001) are preferred because of the lattice structure that matches the graphene lattice. However, 3C polytype structures have also been used on (111)-oriented surfaces in order to maintain a good match with the hexagonal lattice of graphene [161–162].

The SiC wafer is usually precleaned in ultrahigh vacuum (UHV) or in other environments with different techniques in order to increase the graphene quality during graphitization. The three most common techniques are: heating the sample ex situ while hydrogen etching for 30 min at 950 °C; preparing a silicon rich phase (3 × 3) in a Si flux and then heating the sample for 30 min at 1000 °C; or heating the sample at around 1000 °C under Si flux to remove the native oxide and to avoid silicon depletion of the surface [163].

**Figure 28:**
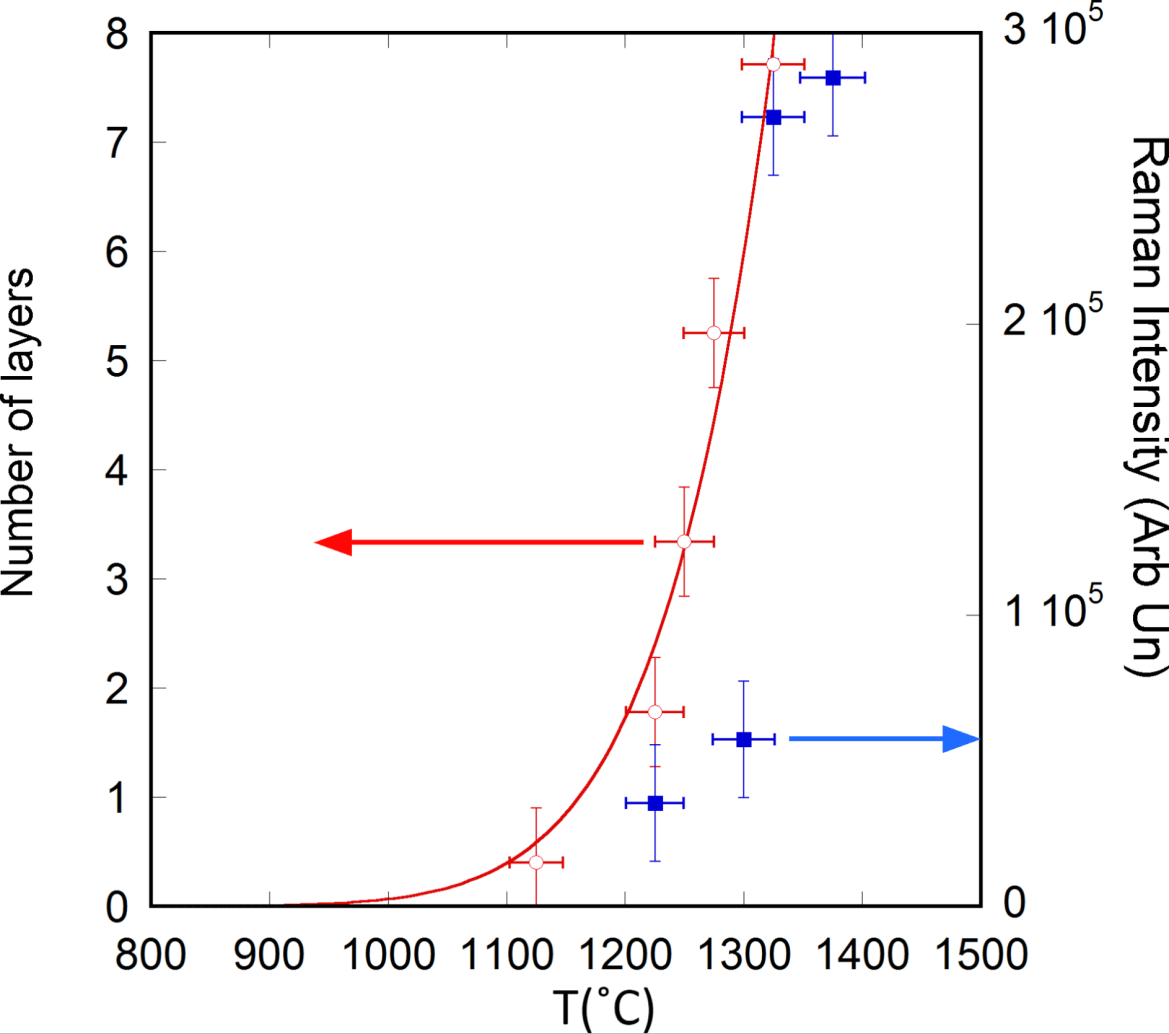
Number of graphene layers grown by annealing 3C-SiC for 10 h in UHV as a function of temperature. Reprinted with permission from [164]. Copyright (2014) Elsevier.

Graphene growth occurs at temperatures of 1200–1350 °C in UHV [164–165] even though graphitic bonds start to appear at temperatures around 1000 °C [166]. Graphene can also be grown at temperatures of 1400–1600 °C in other environments such as inert gas atmosphere [167–168] or in an excess of Si in the gas phase [169] in order to reduce the sublimation rate with the positive pressure. The growth temperature is a very important parameter because it influences the number of graphene layers grown and it is directly related to the Si diffusion (Figure 28) [170].

**Figure 29:**
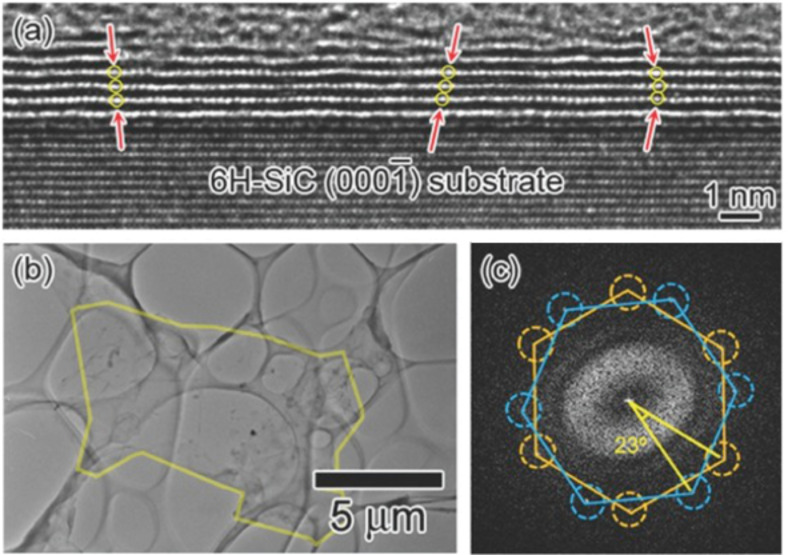
TEM images of MLG on the C-face. (a) A cross-sectional TEM image. (b) A low-magnification TEM image of graphene exfoliated from the SiC substrate. (c) FFT pattern from the area highlighted in (b). Reprinted with permission from [171]. Copyright (2012) Surface Science Society of Japan.

The graphene can also be grown on a C face. The advantages are the absence of a buffer layer and the possibility to easily grow multilayer graphene (MLG) in all directions on the SiC surface (Figure 29a) [171]. The different graphene layers are not stacked in the same direction and are usually rotated about 23° with respect to each other (Figure 29b,c). It has been demonstrated that the rotationally stacked graphene has a van Hove singularity, which generates peaks in the density of the states. This property could be useful to tune the electronic properties of graphene.

**Figure 30:**
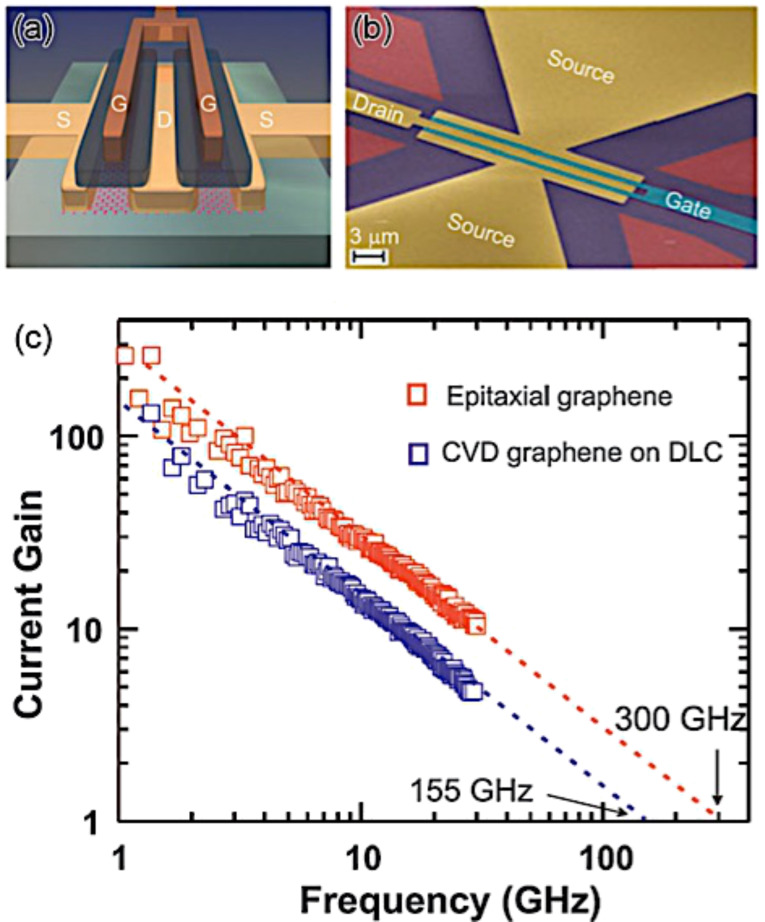
High frequency graphene transistor. (a) and (b) Structure of a graphene-based FET for an analogue radio frequency device. (c) Current gain as a function of frequency, showing a cut-off frequency of 300 GHz for epitaxial graphene on SiC. Reprinted with permission from [173]. Copyright (2012) MRS Bulletin.

One potential application of graphene grown on SiC is for high frequency transistors. Pioneering work was completed in 2006 by Berger et al. [172] by fabricating a field-effect transistor (FET). They were able to show the Dirac nature and the high mobility (25,000 cm^2^ V^−1^ s^−1^) of graphene grown on SiC. An IBM research group recently reached the 300 GHz cut-off frequency for a graphene-based FET grown on SiC. They showed the stable operation of an integrated circuit containing graphene (Figure 30) [173–174].

The two major drawbacks of this graphene synthesis process are the high cost of the SiC wafers and the high temperature involved that is not suitable for the Si technology [81]. The first drawback can been resolved by growing thin layers (100–300 nm) of SiC on top of Si substrates, but further development is required for uniform deposition on a large diameter wafer with a low roughness and elimination of the terraces in order to guarantee high-quality, monolayer graphene. The second drawback could be solved, as in the CVD process, by reducing the growth temperature by the use of PECVD equipment. Industry and research groups worldwide are intensely searching for a solution to this problem because it could contribute to the launch of graphene into the electronic market.

### Organic photovoltaics

**Figure 31:**
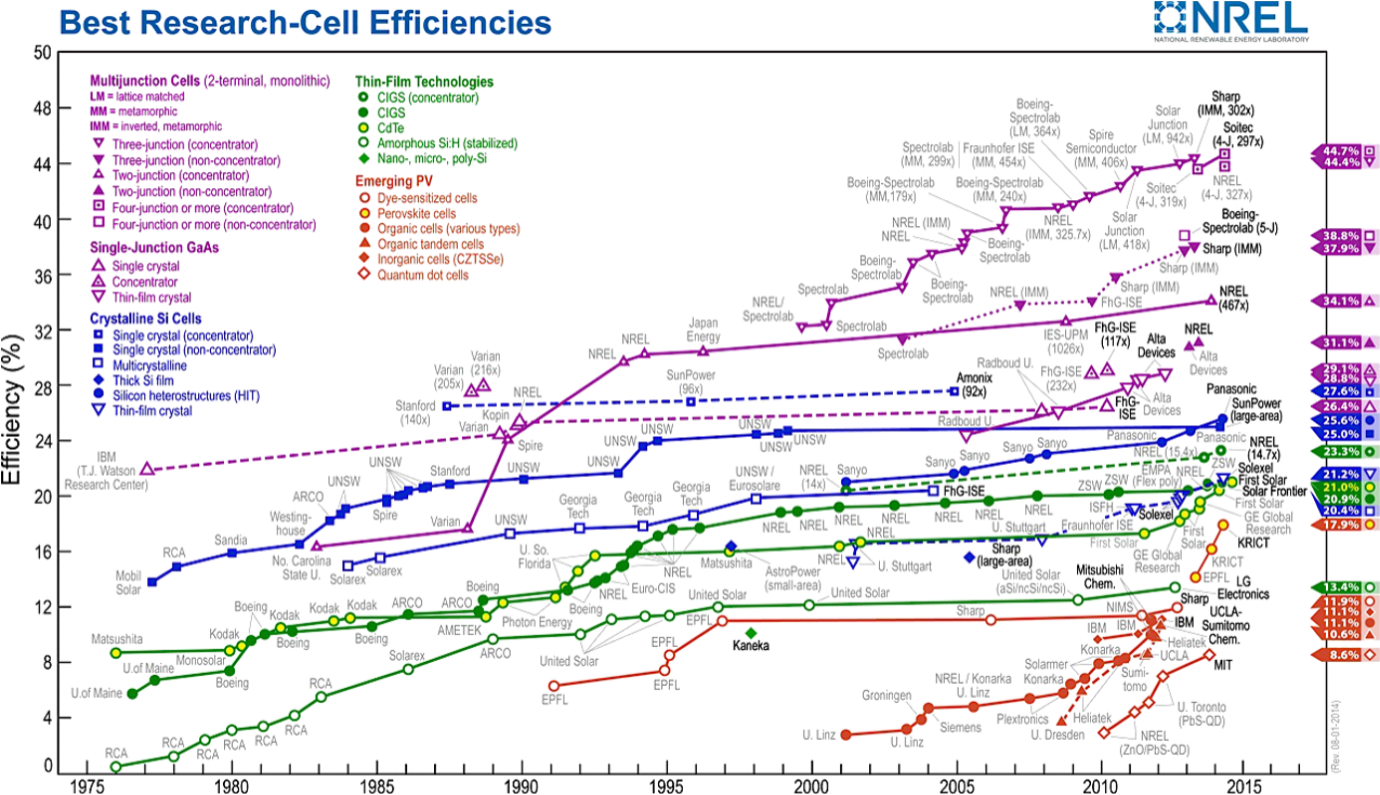
Record solar cell efficiencies, worldwide, as reported by NREL in 2014 [180].

Over the past twenty years, organic photovoltaics have rapidly improved because of the potential to obtain a manufacturing process that is faster, less expensive and with higher production volume as compared to silicon technology [175–178]. Figure 31 shows the best solar cell efficiencies reported by the National Renewable Energy Laboratory (NREL) in the United States over the last 40 years. Of note is that the organic solar cell efficiencies increased from 4 to 12% (record established by Heliatek in 2013 for a tandem, organic solar cell) in just a little over ten years [179].

Three problems still must be solved in order to make this technology competitive with others already present on the market: the power conversion efficiency (PCE) [181], the device lifetime [182] and the large scale production [183].

Typically, an organic solar cell consists of two electrodes (one of which is transparent) and an active layer between them where the generation of free charge carriers occurs. A buffer layer is usually included between each electrode and the active layer in order to prevent charge recombination, which reduces the efficiency of the device. The active layer can be a double layer (one has a stronger affinity for electrons (donor) and the other has a stronger affinity for holes (acceptor)) or a bulk heterojunction (mixture of a donor and acceptor material in bulk). The bulk heterojunction (BHJ) provides a larger volume of paths for the transport of free carriers and more efficient charge separation in comparison to the other structures [184–186]. The first kind of BHJ architecture was originally proposed by Sariciftci et al. [187] and is based on an active blend of a conducting polymer (electron donor material) mixed with fullerene derivatives (electron acceptor material) [188].

**Figure 32:**
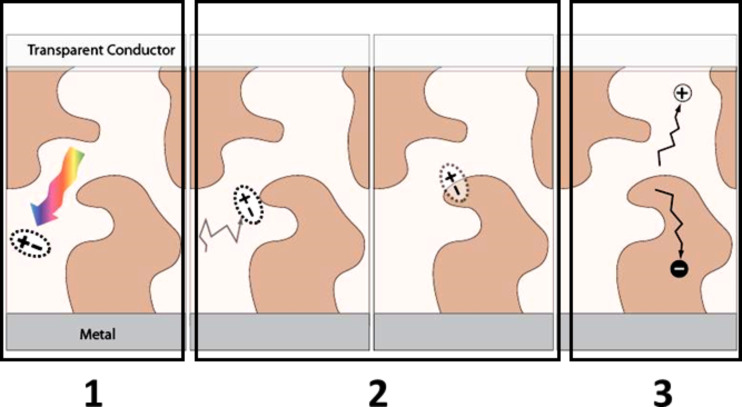
Photocurrent generation steps in an organic solar cell. Step 1: photon absorption in the conducting polymer (donor material). Step 2: creation of an exciton. Step 3: exciton separation at the interface of the heterojunction (interface between the donor and the acceptor).

The generation of a photocurrent due to light incident upon an organic solar cell device consists of three steps (Figure 32) [189–190]:

1. Photon absorption in the conducting polymer (donor material).
2. Creation of an exciton. An exciton is a bound state of an electron and a hole that are attracted to each other by the electrostatic Coulomb force. Its diffusion length is about 10 nm.
3. Exciton separation at the interface between the donor and the acceptor. Because of the built-in electric field at the interface, the electron is transferred to the acceptor and the hole to the donor (creation of the photocurrent).

One problem is the short lifetime of excitons generated by light. Considering that their recombination distance is between 4 and 20 nm [191–192], the morphology of the active layer is an important parameter for the performance of the device [193–195].

#### Fullerene derivatives in organic solar cells

**Figure 33:**
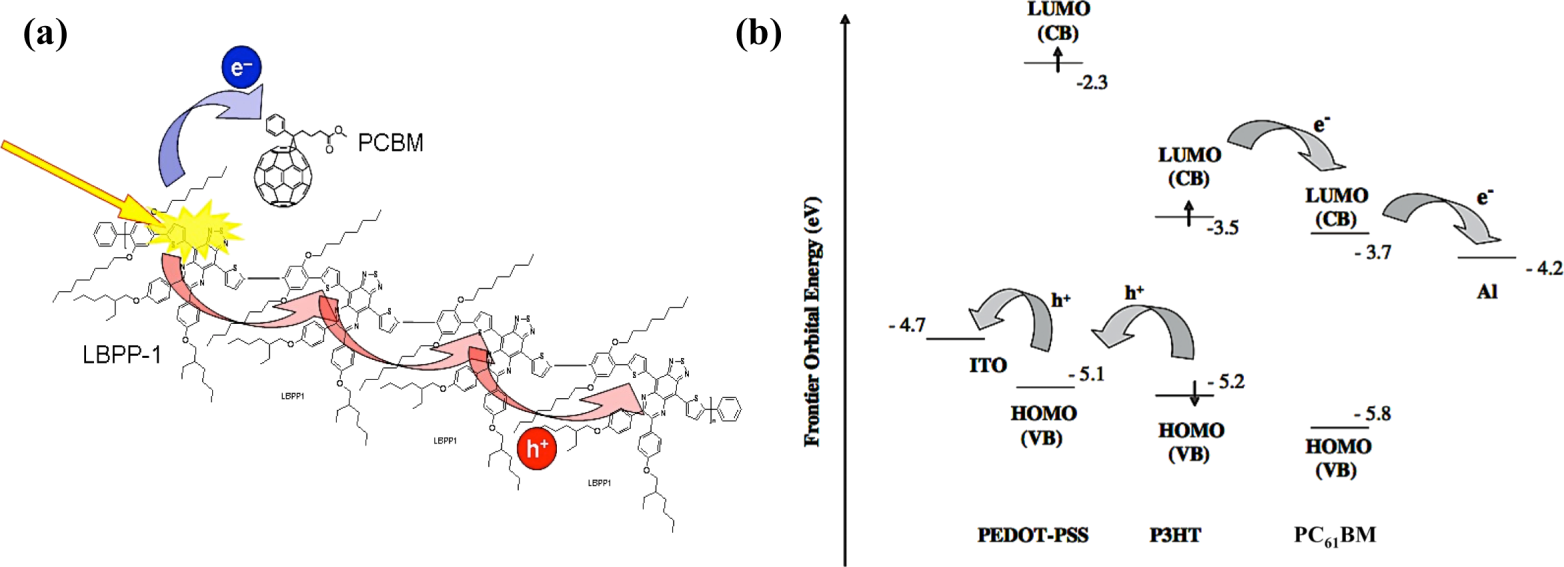
(a) Electron transfer from P3HT to PCBM after generation of the exciton at the interface of the two materials. (b) HOMO and LUMO levels of the different materials in an organic solar cell structure ITO/PEDOT–PSS/P3HT–PCBM/LiF/Al. Reprinted with permission from [26]. Copyright (2005) John Wiley and Sons.

The BHJ solar cells currently most studied are based on a fullerene derivative commonly called [6,6]-phenyl-C_61_-butyric acid methyl ester (PC_61_BM or PCBM) as the acceptor material and the conducting polymer poly(3-hexylthiophene-2,5-diyl) (P3HT) as the donor material [196–198]. Both materials are commercially available and guarantee stable devices. In the regular structure, indium tin oxide (ITO) is typically used as the transparent conducting anode and poly(3,4-ethylenedioxythiophene)/poly(styrene sulfonate) (PEDOT/PSS) as the electron blocking layer. The blend of PCBM and P3HT form the active layer onto which a thin layer of lithium fluoride (LiF), used to block holes, and a layer of aluminium as cathode are coated respectively (Figure 33a) [199–200].

**Figure 34:**
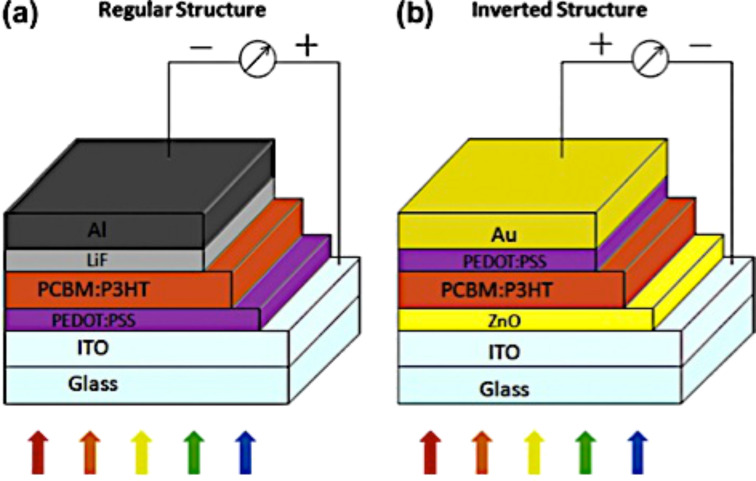
(a) Schematic of a regular organic solar cell structure. (b) Schematic of an inverted organic solar cell structure. Reprinted with permission from [202]. Copyright (2014) Elsevier Limited.

The specific choice of these materials is due to the fact that the exciton separation at the acceptor/donor interface and the transport of the charges across the device is strongly affected by the energy band alignment. Figure 34a shows how, in a regular structure, the electrons and holes can easily move from the donor/acceptor interface to the respective electrodes because the energy values of the lowest unoccupied molecular orbital (LUMO) and the highest occupied molecular orbital (HOMO) of each material are very similar [26]. An organic solar cell device can also have a reversed structure by inserting a hole blocking layer between the transparent electrode and the active layer in order to collect electrons and an electron blocking layer on the metallic electrode in order to collect holes. Figure 34b shows a typical inverted structure composed of ITO as the cathode, zinc oxide (ZnO) as the hole blocking layer, PC_61_BM/P3HT as the active layer, PEDOT/PSS as the electron blocking layer and gold (Au) as the anode [201].

**Figure 35:**
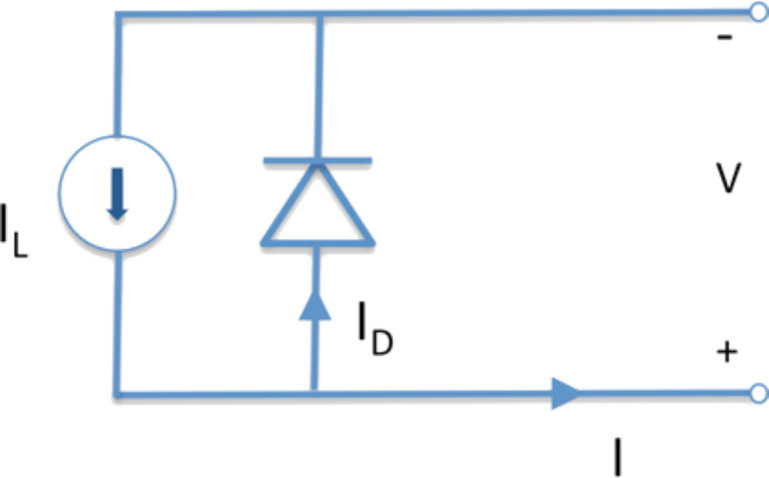
Simple equivalent circuit for a solar cell.

In order to characterize the performance of an organic solar cell, the electrical current (*I*) and the voltage potential (*V*) across the device are measured and plotted on the *y*- and *x*-axis, respectively, under a standard illumination. The current produced by a solar cell is the combination of the current of the solar cell diode (*I*_D_) in the dark with the light-generated current (*I*_L_) (Figure 35).

The total current calculated from the circuit in Figure 35 is [203]:

[2]
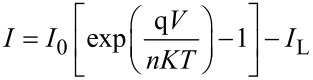


**Figure 36:**
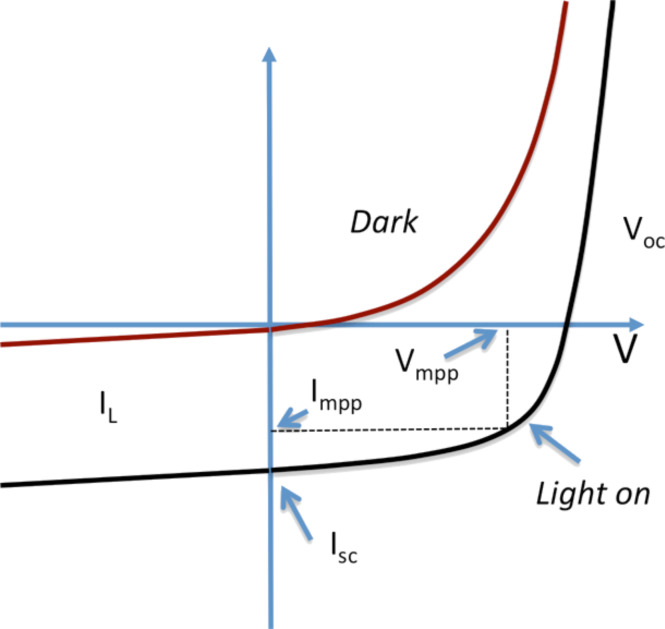
*I*–*V* curves of a solar cell. *I*_L_ indicates the current under illumination. *V*_oc_ and *I*_sc_ represent the open circuit voltage and the short circuit current, respectively, while *V*_mp_ and *I*_mp_ indicate the maximum power point.

The *I*–*V* curve of a solar cell is the superposition of the *I*–*V* curve of the solar cell diode in the dark, described by the Shockley diode equation, with the light-generated current. The light has the effect of shifting the *I*–*V* curve down into the fourth quadrant so that power can be extracted (Figure 36).

**Figure 37:**
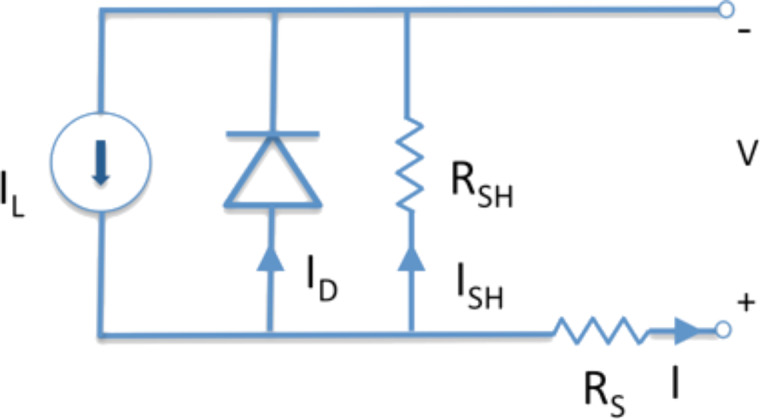
Detailed equivalent circuit for a solar cell.

Typical resistive effects are unfortunately present and they contribute to reduced performance of the device. The most common parasitic resistances are series resistance (*R*_S_) and shunt resistance (*R*_SH_). *R*_S_ and *R*_SH_ are included in a more complicated equivalent circuit model (Figure 37).

The efficiency, η, of a solar cell can be calculated from the *I*–*V* curves (as in Figure 36) [203]:

[3]
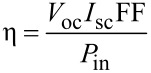


where *V*_oc_ is the open circuit voltage, *I*_sc_ the short circuit current, *P*_in_ is the input power and FF is the fill factor that indicates the “squareness” of the *I*–*V* curve. The FF can also be calculated from Figure 36 [203]:

[4]
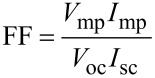


where *V*_mp_ and *I*_mp_ correspond to the maximum power point on the *I*–*V* curves.

The previously described, remarkable properties of carbon nanomaterials make them very attractive for use in organic photovoltaics [16]. Fullerenes (C_60_) were the first to be proposed because they have a response typical of n-type semiconductors and are able to accept electrons coming from the photoexcitation of a conducting polymer [204]. The first heterojunction based on C_60_ was realized in 1993 [205]. Unfortunately, the performance of the device was limited by the fact that C_60_ could not be well-dispersed and could not penetrate into the conducting polymer. For this reason, C_60_ derivatives were proposed because of their ability to diffuse into the polymer film and to form an intermixed layer. In particular, 1-(3-methoxycarbonyl)propyl-1-phenyl[6,6]methanofullerene or the PC_61_BM derivative is more soluble in organic solvents than pristine C_60_ [35]. Currently, many stable devices are prepared with a mixture of different conducting polymers and PC_61_BM, achieving a stable PCE of about 4% [206].

**Figure 38:**
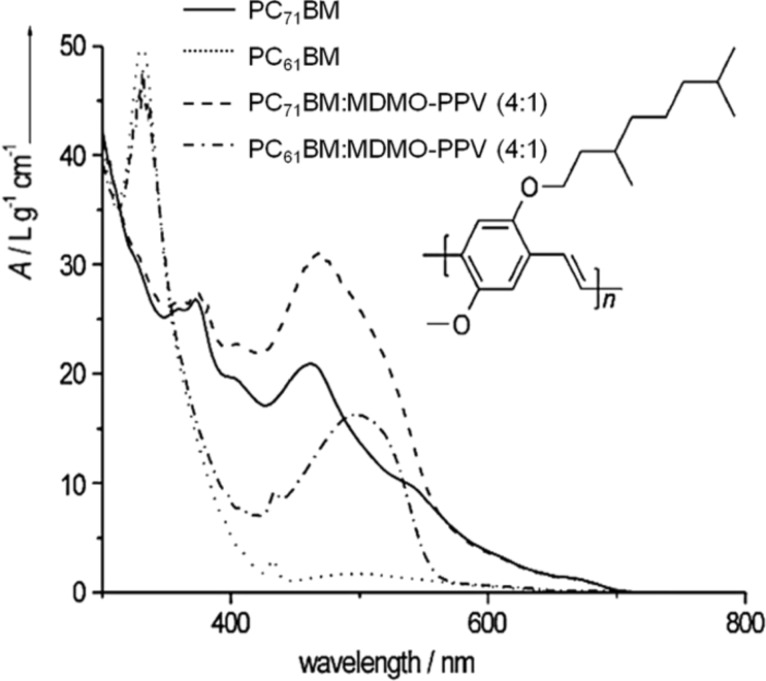
UV–vis spectra of PC_71_BM and PC_61_BM, both in toluene. To illustrate the contribution of MDMO-PPV to the absorption, the (normalized) spectra of PC_71_BM/MDMO-PPV and PC_61_BM/MDMO-PPV, also in toluene, are presented. The inset shows the structure of poly(2-methoxy-5-[3’,7’-dimethyloctyloxy]-p-phenylene vinylene) (MDMO-PPV). Reprinted with permission from [207]. Copyright (2015) John Wiley and Sons.

Because PC_61_BM has small absorption peaks, other fullerene derivatives such as phenyl-C_71_-butyric acid methyl ester (PC_71_BM) have recently been used to provide better absorption in the visible spectra [207], boosting the PCE to values higher than 7% (Figure 38) [208].

Other fullerene derivatives and C_60_-functionalized macromolecules have also been proposed for the preparation of all-polymer type solar cells; however, the performance to date was too low due to the presence of large solubilizing groups that decrease the charge transport [209].

#### Carbon nanotubes in organic solar cells

**Figure 39:**
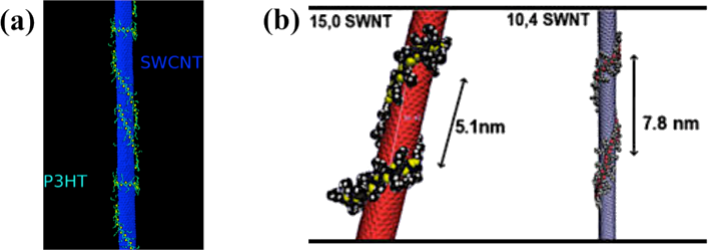
(a) Molecular dynamics simulations of P3HT wrapped around a SWNT (15,0). Reprinted with permission from [214]. Copyright (2010) American Chemical Society. (b) Helices form on (15,0) and (10,4) SWNTs during the folding of P3HT with orthogonal initialization. The chirality may affect the pitch distance to some extent. Reprinted with permission from [213]. Copyright (2010) American Chemical Society.

**Figure 40:**
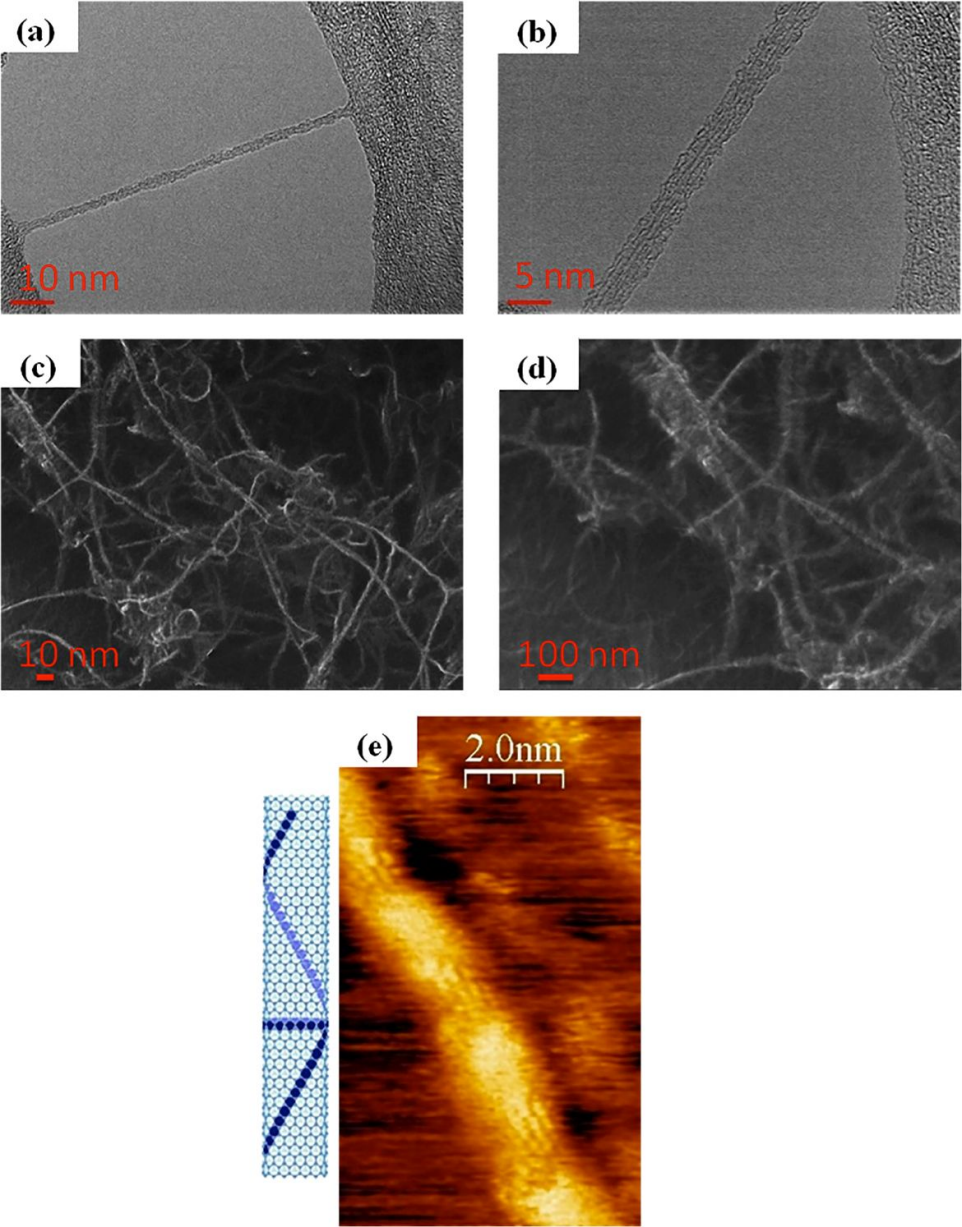
(a) and (b) TEM images of P3HT wrapping around a SWNT (7,6) (images taken at QUT, not yet published). (c) and (d) SEM images of P3HT wrapping around bundles of SWNTs (7,6) (images taken at QUT, not yet published). (e) STM image and schematic of P3HT wrapping around a SWNT (15,0). Reprinted with permission from [218]. Copyright (2009) AIP Publishing LLC.

CNTs have also been used in organic solar cells to replace fullerenes as the acceptor material. Because of their high aspect ratio, electrical conductivity, and tunable optical and electronic properties, the necessary quantity of CNTs introduced into the device could be significantly lower as compared to the amount of required PCBM [210]. In fact, the ratio of PCBM and P3HT in the blend is usually 1:1. However, for CNTs, it could be much lower (≈3 wt % of the P3HT) [211]. CNTs are either partially [212] or completely [211] replacing the PCBM compounds in organic solar cells. For example, when they are mixed with P3HT, the polymer chains tend to wrap around the CNTs with an electron transfer between the CNTs and the P3HT as demonstrated in Figure 39 [213–214] and experimentally observed in SEM and TEM images (Figure 40) [215]. This interaction is usually stronger if the SWNTs are semiconducting instead of metallic [216–217]. Heterojunctions between SWCNTs and the P3HT molecules that have been studied by means of scanning tunneling microscopy and computer simulation open possibilities for making novel solar cells [218–219]. Devices were made by blending CNTs with P3HT, resulting in a maximum efficiency of 3.36% [220]. However, by blending n-doped MWCNTs (n-MWCNTs) into a mixture of PTB7 and PC_71_BM, the device reaches an efficiency as high as 8.6%. It was concluded that the incorporation of N-MWCNTs leads not only to increased nanocrystallite size but also smaller phase-seperated domain sizes of both PTB7 copolymers and PC_71_BM. n-MWCNTs serve as both exciton dissociation centers and charge transfer channels. p-type SWCNTs can be easily obtained by chemical doping. A common method is the nitric acid treatment of CNTs, which introduces nitrogen dopants as well as an increase in the electrical conductivity [221–222]. The PEDOT/PSS can cause degradation of the active layer and the ITO electrode because of its hygroscopicity and acidity, which results in a decrease in the lifetime of the solar cell device [223]. Hence, the network film of p-type SWCNTs has been used to replace the PEDOT/PSS buffer layer in organic solar cells, with a device structure of ITO/SWCNTs/P3HT–PCBM/Al [223–224]. Using the SWCNT film as the hole transport layer, the energy conversion efficiency of the organic solar cell is equivalent to that of the component device with PEDOT/PSS.

Efforts have also been made toward the development of hybrid solar cells with a p-CNT/n-Si structure. By coating a transparent p-type SWCNT network film onto a Si substrate, the solar cell is formed and the energy conversion efficiency can be >11% [225]. By coating a layer of MoO*_x_* onto the CNT film, the efficiency reaches 17% [226].

In fact, CNTs can be grown directly on the ITO substrate [227–228]. In device fabrication, the mixture of P3HT and PCBM is coated onto the substrate, and CNTs are used as 3D electrodes to collect charges from the active media. The problem is that MWCNTs grown at relatively low temperature onto the ITO glass are tens of nanometers thick and have low areal density. With such a substrate, the energy conversion efficiency of organic solar cells is lower than 1%. The best result so far, i.e., 2.1% energy conversion efficiency, was obtained in 2013 by growing CNTs onto fluorine-doped tin oxide (FTO) glass, which is more resistant to high temperature than ITO [229].

Other research groups are focusing their work on replacing ITO, which is expensive and brittle, with a carpet of CNTs in bundles because of their optical transparency, high conductivity properties and the potential to be deposited onto a flexible substrate [230–231].

#### Graphene and derivatives in organic solar cells

**Figure 41:**
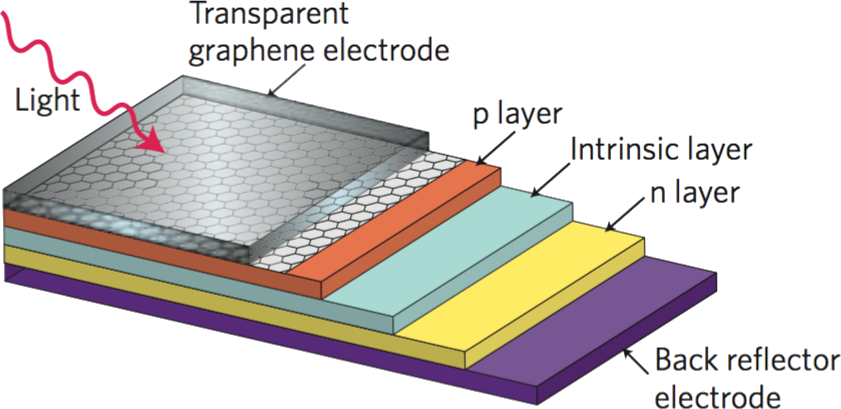
Schematic of an organic solar cell with a transparent graphene electrode. Reprinted with permission from [234]. Copyright (2010) Nature Publishing Group.

More recently, graphene and its derivatives have also been proposed for organic solar cells. In particular, pristine graphene can be used as a transparent electrode similar to the CNT carpet (Figure 41) [232–234].

Graphene is not typically found in the active or buffer layers because of its zero band gap structure, but the introduction of functional groups could open up more possibilities of graphene integration into different layers of an organic solar cell device. On the other hand, GO can easily be integrated into organic solar cells because of its semiconducting behavior that can be finely tuned as a function of the degree of oxidation.

**Figure 42:**
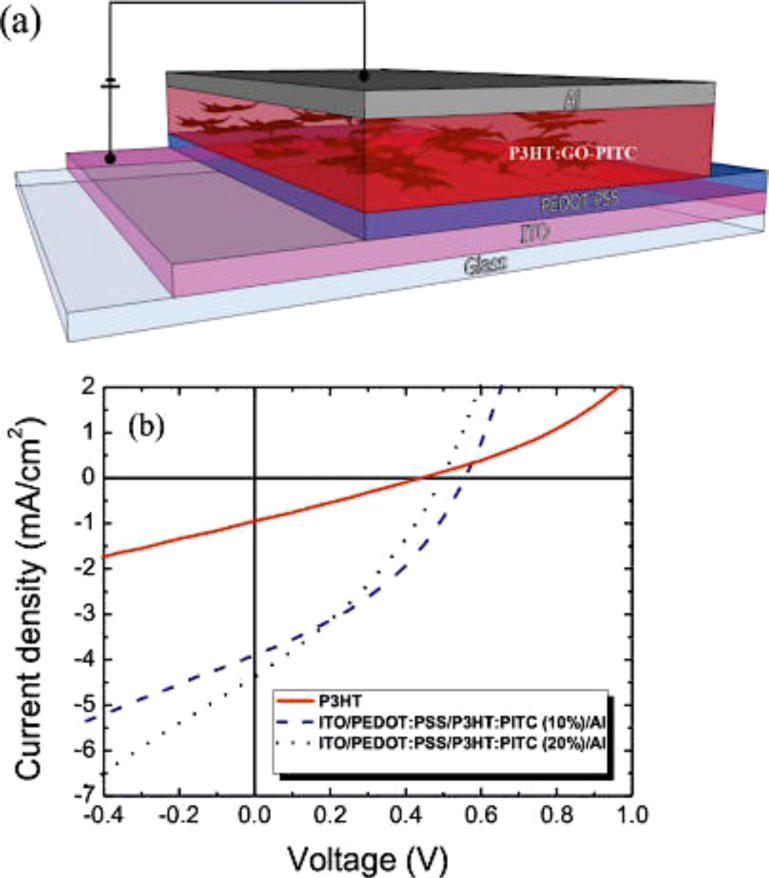
(a) Schematic of a photovoltaic device with a P3HT/GO–PITC thin film as the active layer and the structure ITO–PEDOT/PSS(30 nm)–P3HT/GO–PITC(110 nm)/Al(80 nm). (b) Experimental *I*–*V* curves of the photovoltaic devices based on P3HT (red curve) and P3HT/GO–PITC composites (dashed curve, 10 wt %; dotted curve, 20 wt %) after post fabrication thermal annealing at 160 °C for 20 min. Reprinted with permission from [236]. Copyright (2012) American Chemical Society.

GO has been employed in the active layer for the effective exciton charge separation and charge transport when mixed with a conducting polymer, such as P3HT. This is due to the large surface interface area of the acceptor/donor and continuous pathway, similar to CNTs [235–237]. In order to increase the solubility in typical organic solvents (e.g., dichlorobenzene) used to disperse the conducting polymers, the GO can be functionalized with other compounds such as phenyl isothiocyanate (PITC) (Figure 42) [236].

**Figure 43:**
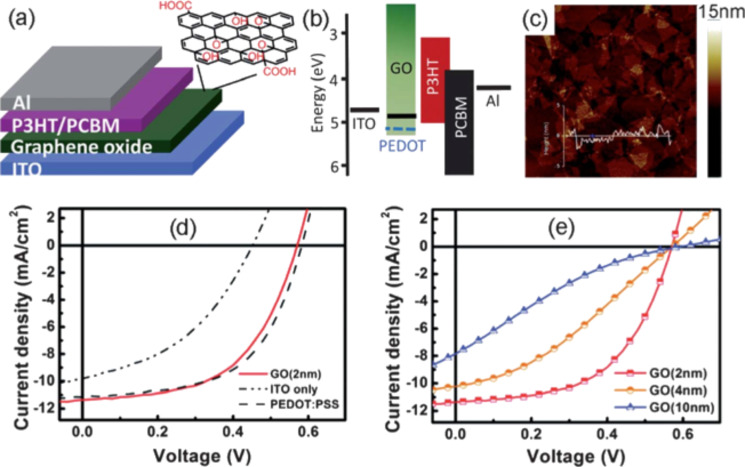
(a) Schematic illustration of a device structure with GO as the buffer layer. (b) Energy level diagrams of the ITO bottom electrode, interlayer materials (PEDOT/PSS, GO), P3HT (donor), PCBM (acceptor), and the Al top electrode. (c) An AFM height image of a GO thin film with a thickness of approximately 2 nm. (d) Current density–voltage (*J*–*V*) characteristics of the devices with no hole extraction layer (HEL), with a 30 nm PEDOT/PSS film, and with a 2 nm GO film. (e) *J*–*V* characteristics of the ITO/GO/P3HT–PCBM/Al devices with GO layers of different thicknesses. Adapted from [240]. Copyright (2010) American Chemical Society.

GO has been largely used in the organic solar cell field as a buffer layer. Specifically, it could be a valid candidate to replace PEDOT/PSS as the electron blocking layer because it presents work function values of 4.6–4.8 eV, which are very similar to PEDOT/PSS (Figure 43) [238–241].

**Figure 44:**
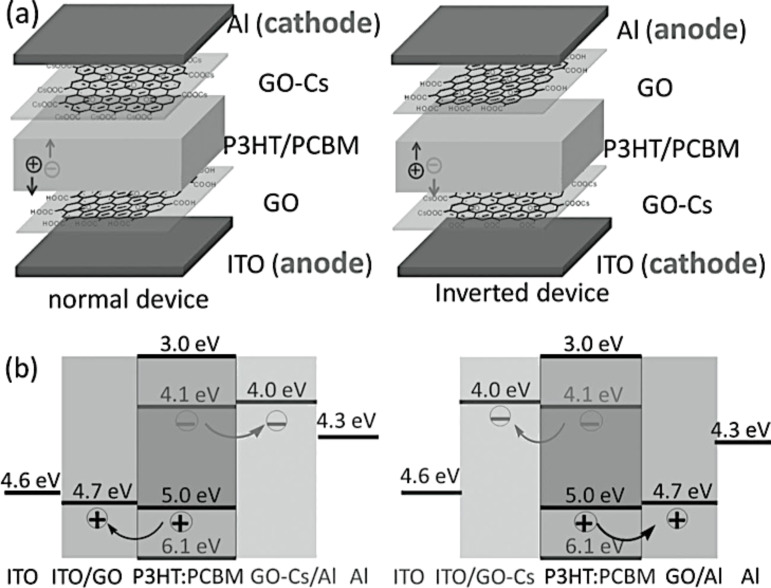
Device structures (a) and energy level diagrams (b) of the normal device and the inverted device with GO as the hole-extraction layer and GO/Cs as the electron-extraction layer. Reprinted with permission from [238]. Copyright (2012) John Wiley and Sons.

When chemically modified with specific dopants (such as caesium (Cs) atoms), graphene could also be used as an electron-blocking layer because its work function can be reduced to 3.9–4.1 eV (Figure 44) [238,241].

**Figure 45:**
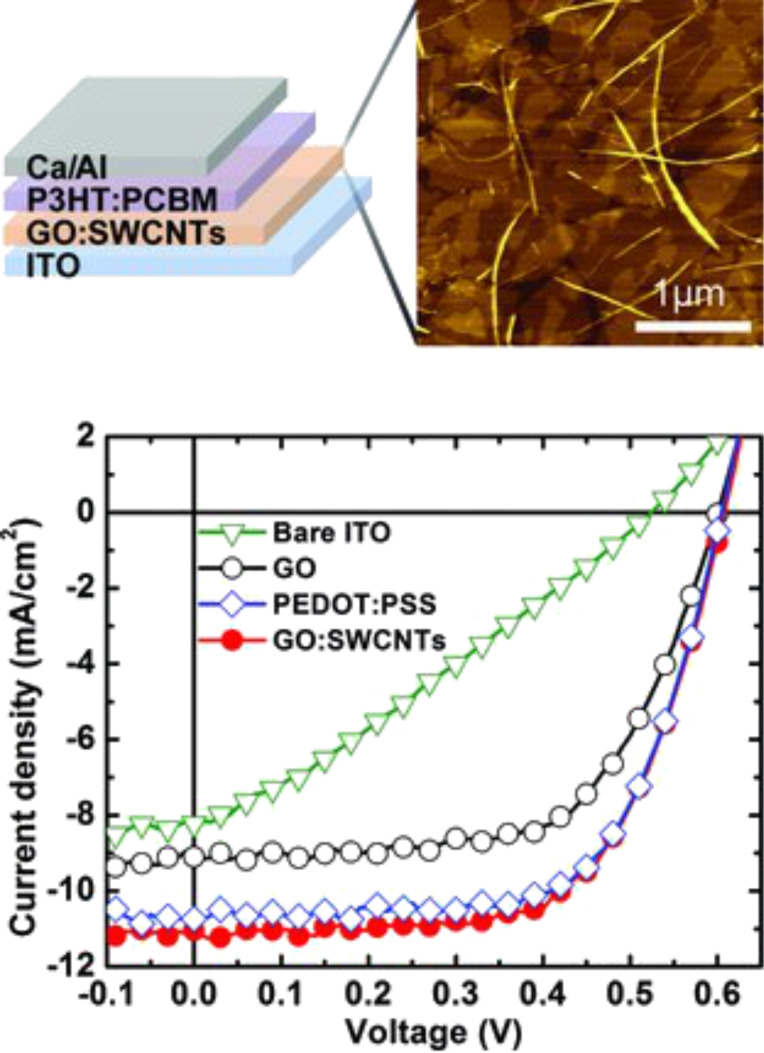
Addition of a small amount of SWCNTs into the GO buffer layer can increase the FF and JSC of devices with GO. Reprinted with permission from [242]. Copyright (2011) John Wiley and Sons.

Because of the high sheet resistance of GO, the addition of SWNTs into the blend can decrease the through-thickness resistance of the GO film by an order of magnitude if used as a buffer layer (Figure 45) [242].

**Figure 46:**
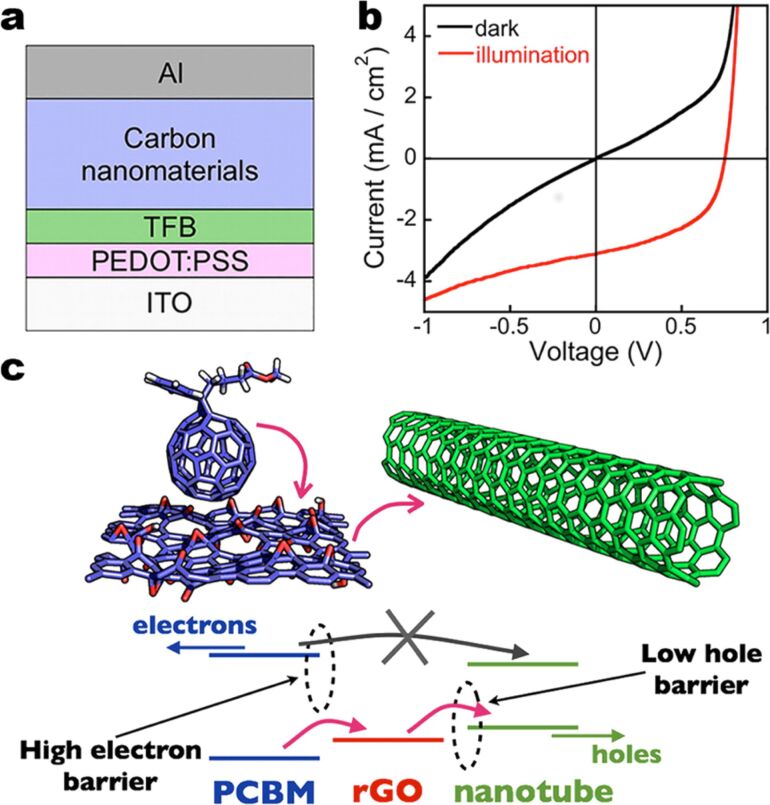
(a) Structure of carbon solar cells where TFB and PEDOT/PSS are the electron-blocking and hole-conducting layers, respectively, deposited on top of the ITO. For the best device efficiency, a blend of rGO, PC_71_BM, and s-SWCNT with a diameter of 1.2–1.7 nm was used as the active layer, which is denoted by “carbon nanomaterials”. (b) Current–voltage curves in the dark and under simulated sunlight illumination of the device. (c) Interface of PC_71_BM/rGO sheet/s-SWCNT. Hole carriers photogenerated within PCBM are transferred to rGO due to a large Schottky barrier for electrons, as shown by pink arrows, and then to s-SWCNT. Reprinted with permission from [243]. Copyright (2012) American Chemical Society.

Bernardi et al. [243] were the first to demonstrate the possibility to have a solar cell comprised of only carbon nanomaterials in the active layer without the use of any conducting polymer. In their work, the active layer was composed of only PC_71_BM, semiconducting SWNTs, and reduced GO, achieving a PCE of 1.3%. They also used ab initio calculations to demonstrate efficiency limits of up to 13% for this device, which is comparable to those predicted for polymer solar cells (Figure 46).

**Figure 47:**
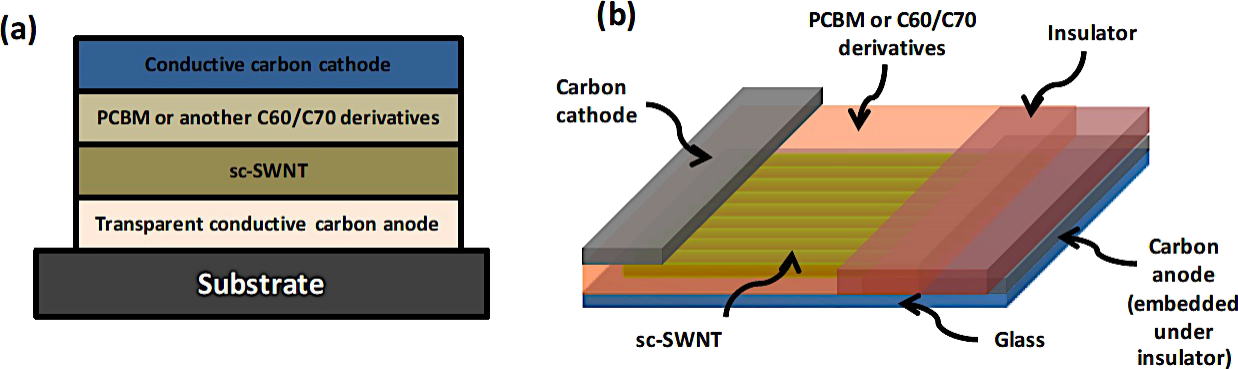
Schematic of the two basic all carbon nanomaterial-based solar cell device structures: (a) a typical vertical structure. An inverted vertical structure is also possible where the conductive cathode will be in contact with the substrate. (b) A horizontal structure that will take advantage of aligned SWNTs or aligned C_60_ microribbons. Reprinted with permission from the Global Climate & Energy Project, Stanford University [244].

In 2011, a research group at Stanford University proposed for the first time a solar cell based entirely on carbon nanomaterials in two architectures, one vertical and one horizontal (Figure 47) [244].

In 2012, the same group fabricated and tested a complete carbon-based solar cell device. Although it achieved a PCE of only 5.7 × 10^−3^%, they were the first worldwide to demonstrate an efficiency from a device composed solely of carbon nanomaterials [245]. A transparent electrode based on rGO was used as the anode and n-type-doped SWNTs as the cathode. Sunlight was absorbed by the semiconducting SWNTs to generate excitons that were split in the active layer composed by SWNTs and PC_61_BM [245].

To date, the PCE is still limited by the low photon absorption of the active layer. New compounds are being used in the active layer or novel carbon nanomaterials, such as fullerene derivatives and SWCNTs, and new architectures have been implemented. Although carbon nanomaterials are strong light absorbers, their percentage in the active layer is optimized to achieve the maximum PCE. Also, the thickness of the active layer is usually less than 1 µm because increased thickness is unfavorable for exciton extraction. At present, tandem organic solar cells based on the combination of high band gap and low band gap polymers represent a reliable way to achieve improved spectral range for the photoabsorption in the device. However, the challenge of trapping more incoming photons in terms of light-electron conversion [246] has not been solved.

Graphene has also been employed in solar cells other than the organic ones. By coating CVD graphene onto n-type Si, Schottky junction solar cells with efficiencies up to 1.5% were made by Li et al. [247]. The efficiency of this type of solar cell was increased to 8.6% under AM 1.5 illumination by chemically doping the graphene sheets with bis(trifluoromethanesulfonyl)amide ((CF_3_SO_2_)_2_NH) [248]. By doping the CVD graphene film coated onto n-Si with HNO_3_, and subsequently spin coating a layer of colloidal TiO_2_ antireflection film, Shi et al. improved the solar cell efficiency to 14.5% under standard illumination [249]. Graphene films in dye-sensitized solar cells are mainly used as counter electrodes, which outperform platinum electrodes in some cases [250–252].

### Supercapacitors

Electrochemical capacitors (ECs) have been known by different names such as “ultracapacitors” or “power capacitors” but the most recognized name today is “supercapacitors”. The term supercapacitor was introduced by NEC because it was the first company to commercialize a device with the name SuperCapacitor^TM^ in 1971 [253].

Supercapacitors have been in development since 1957 when Becker [254] first used carbon flooded with a sulfuric acid electrolyte to develop charge storage at the interface between these two materials. However, it was not until 1969 that the company SOHIO [255] first launched this technology into the market. The real success of supercapacitors started in the 1990s when government programs in the United States began giving funds for this technology to be incorporated into hybrid vehicles for providing necessary power for acceleration [256].

**Figure 48:**
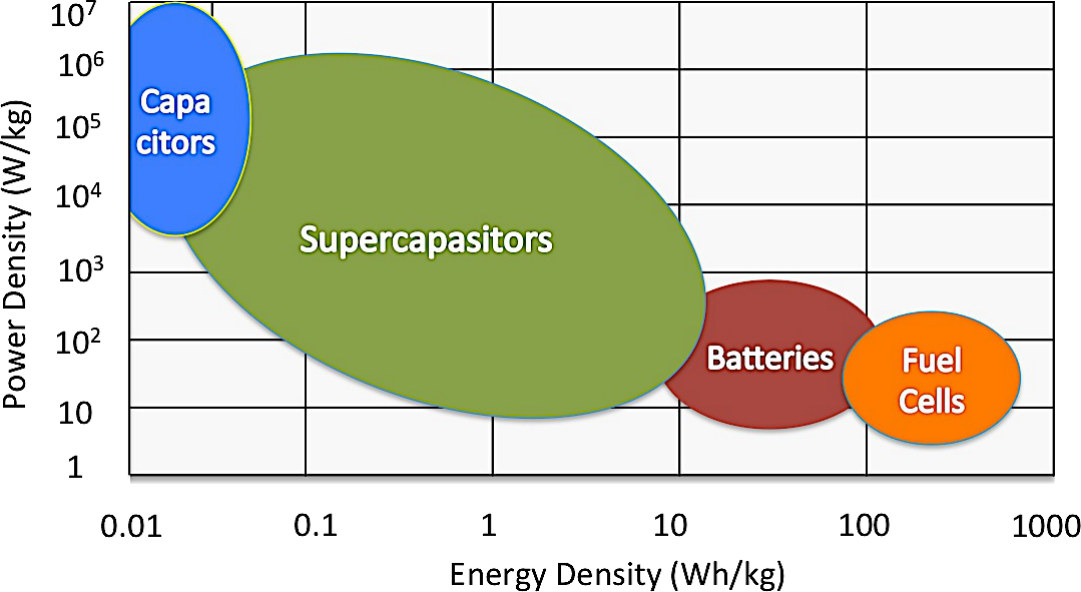
Energy density vs power density (Ragone plot) for various energy storage devices [257].

Supercapacitors can provide a higher power density but a smaller energy density compared to traditional chemical batteries, which make them very attractive for applications where instantaneous power is required. The other key characteristics of supercapacitors are: ability to charge–discharge within seconds; a long lifetime of more than 10^6^ cycles; environmentally friendly; and stable operation at various temperatures. Figure 48 shows a typical energy density vs power density plot, also called a Ragone plot, that compares different energy storage devices. It can be seen that supercapacitors fill the gap between capacitors and batteries [257].

Today, several companies such as Maxwell, FastCap Systems, NEC, Panasonic, Tokin and even car companies such as Volvo are investing further in the development of this technology because of the potential large amount of energy in a small component that can be easily integrated into a device. Volvo for example is working on reducing the weight and increasing the space in a hybrid vehicle by incorporating supercapacitors in the frame of the car [258].

**Figure 49:**
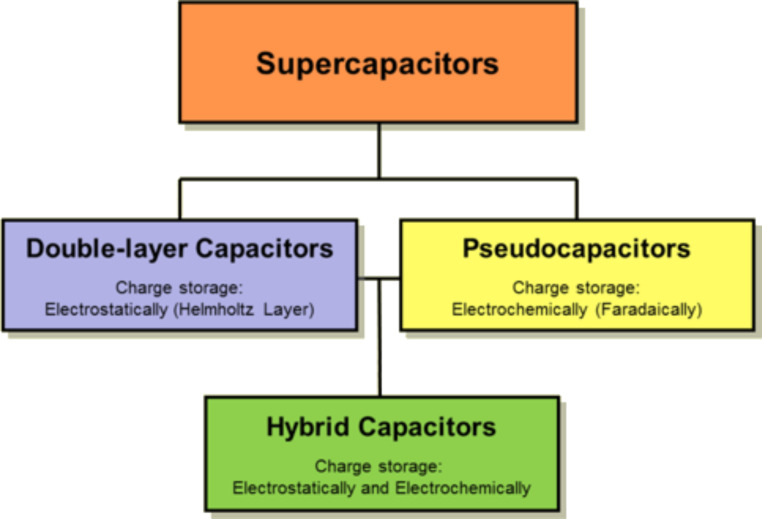
Hierarchical classification of supercapacitors and related types [259].

Supercapacitors are typically divided in two categories: electric double-layer capacitors (EDLCs) and pseudo-capacitors. A subcategory called hybrid capacitors can be identified if the EDLCs and the pseudo-capacitors are combined together into a single device (Figure 49) [253].

**Figure 50:**
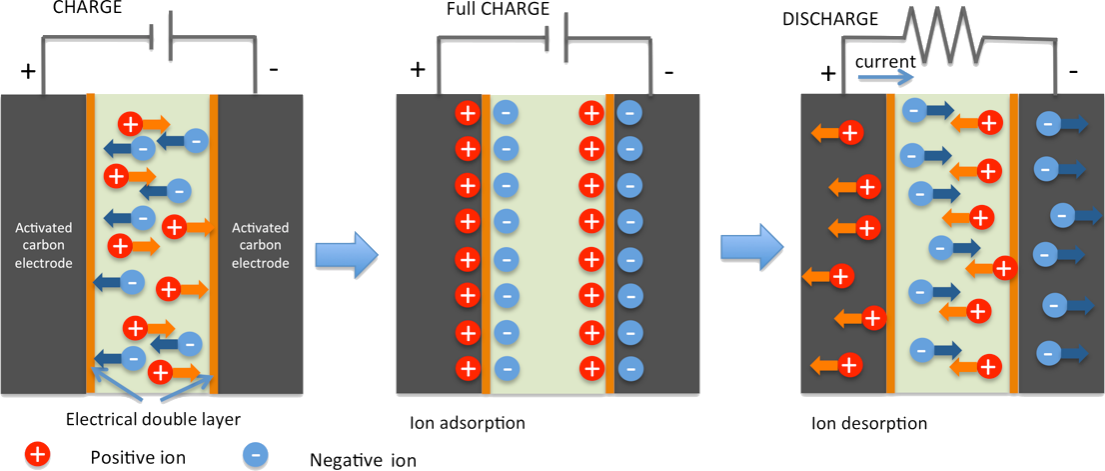
Charge and discharge processes of an EDLC.

EDLCs typically store the energy at the electrode/electrolyte interface as shown in Figure 50. During the charging phase, an external electric field applied to the device moves the ions at the electrode/electrolyte interface [253].

Positive and negative ions are accumulated at this interface, typically on the order of 5–10 Å [260]. The conventional equation that defines the capacitance is [203]:

[5]
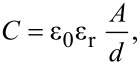


where ε_0_ is the permittivity of the vacuum, ε_r_ is the permittivity of the electrolyte and *d* is the thickness of the double layer with surface area *A*. The thickness (*d*) of the interface is very small (on the order of Å), as discussed previously, while the surface area (*A*) of the electrode is usually very due to the choice of porous structures with a large internal surface area usually chosen for supercapacitor applications. In this way, the capacitance can reach a high value (>10 μF/cm^2^).

**Figure 51:**
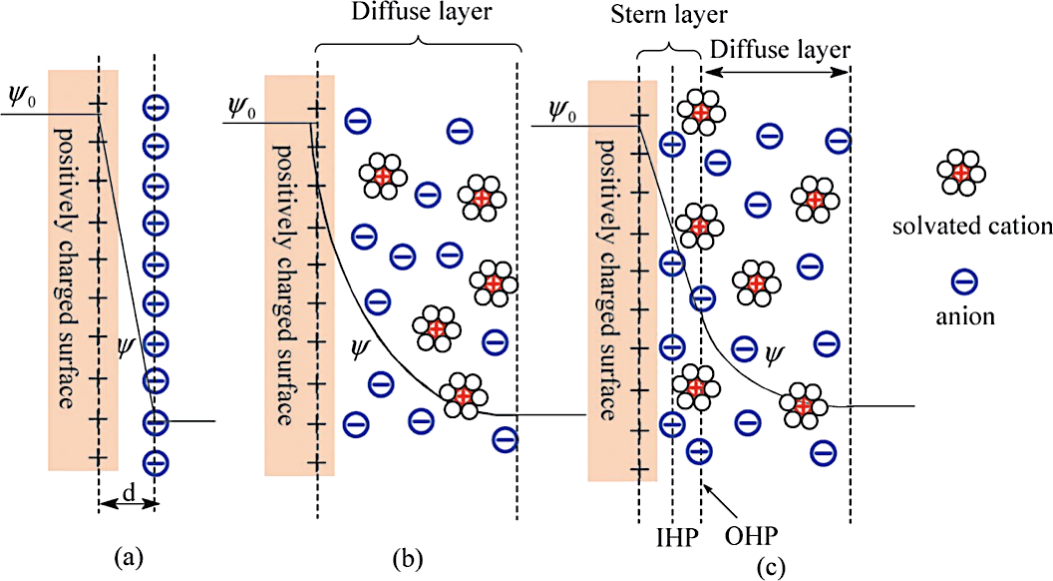
Models of the electrical double layer at a positively charged surface: (a) the Helmholtz model, (b) the Gouy–Chapman model, and (c) the Stern model, showing the inner Helmholtz plane (IHP) and outer Helmholtz plane (OHP). The IHP refers to the distance of closest approach of specifically adsorbed ions (generally anions) and OHP refers to that of the nonspecifically adsorbed ions. The OHP is also the plane where the diffuse layer begins. *d* is the double layer distance described by the Helmholtz model. Ψ_0_ and Ψ are the potentials at the electrode surface and the electrode/electrolyte interface, respectively. Reprinted with permission from [265]. Copyright (2009) Royal Society of Chemistry.

The model described to store the charge at the electrode/electrolyte interface was first derived by Helmholtz [261] in the 19th century. But only in around 1910 Gouy [262] and Chapman [263] were able to expand the model by considering the thermal motion of the electrolyte ions that lead to a diffuse layer. In 1924, Stern [264] combined the two theories in order to identify an inner plane, inner Helmholtz plane (IHP), and an outer plane, outer Helmholtz plane (OHP). The ions of the IHP are strongly bound to the IHP resulting in a strong electric field in that area (Figure 51) [265]. The capacitance established at one electrode will be given by the sum of a compact double layer capacitance (*C*_H_) and diffusion region capacitance (*C*_diff_):

[6]
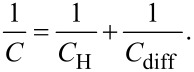


The ideal total capacitance in an EDLC is given by the sum of the capacitances established at the two electrode/electrolyte interfaces [266]:

[7]
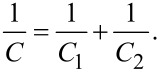


**Figure 52:**
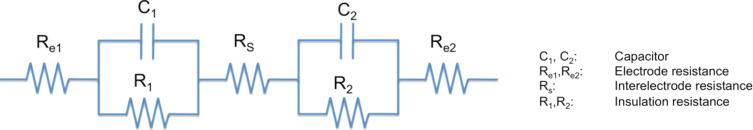
Simple equivalent circuit.

Unfortunately, other parameters have to be considered in a simple equivalent circuit of an EDLC, such as the insulation resistances (*R*_1_, *R*_2_), the electrode resistances (*R*e_1_, *R*e_2_) and the interelectrode resistance (*R*_S_) [253] (Figure 52).

In contrast, pseudo-capacitors are devices where the charge is not stored electrostatically but electrochemically, similar to what happens in conventional lithium ion batteries. The materials that compose the electrode are subjected to a faradaic oxidation/reduction reaction at specific potentials during charging and discharging processes involving absorption or intercalation with the electrolyte. Pseudocapacitive materials such as conducting polymers (e.g., polyanaline (PANI) [267]) or metal oxides (e.g., ruthenium oxide (RuO_2_) [268]) can have 10–100 times larger capacitance than EDLCs but they suffer from poor stability, short lifetime and are expensive to synthesize. Because of these drawbacks, they are usually combined with carbon materials creating hybrid supercapacitors. The capacitance for a pseudocapacitor is calculated using the following equation [253]:

[8]
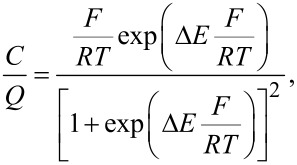


by using the Nernst equation, which describes the general oxidation/reduction phenomenon. Here, *E* represents the equilibrium potential for the reaction and Δ*E* = (*E* – *E*^0^), *F* is the Faraday constant defined as the number of coulombs per mole of electrons and *Q* describes the charge related to the materials subjected to oxidation or reduction.

Pseudocapacitors are not explored in this work because of the drawbacks previously described and because of the uncertainty of whether they should be categorized in the supercapacitor or in the battery family. In fact, their operating mechanism is more similar to a chemical battery than to a supercapacitor.

In order to characterize the electrical properties of a supercapacitor, three electrochemical measurement techniques are usually performed: cyclic voltammetry (CV), galvanostatic charging/discharging and electrochemical impedance measurements.

**Figure 53:**
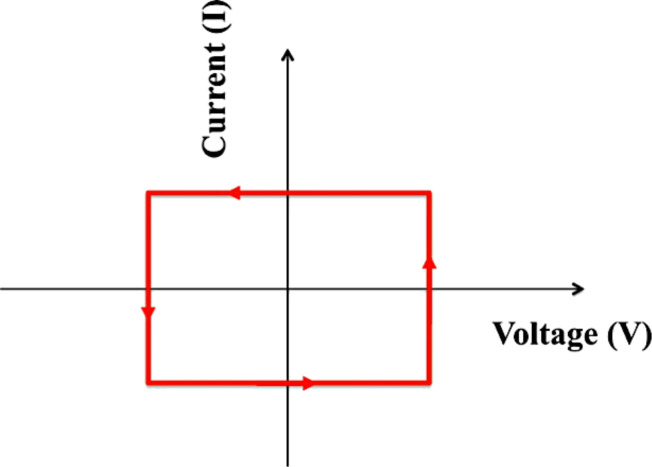
CV curve of an ideal supercapacitor.

The CV technique consists of applying a potential sweep rate d*V*/d*t* from a lower limit to an upper limit and vice versa in order to measure the reversibility of the process and the stability of the device. The characteristic of the CV curve depends on the rate of the electron transfer reactions, the chemical reactivity of the electrode/electrolyte and the voltage scan rate [269]. The CV measurement is usually plotted as voltage (*V*) vs current (*I*) with an ideal supercapacitor presenting a rectangular CV curve when the capacitance (*C*) is constant across the different potential scan range (Figure 53). The capacitance (*C*) can be calculated by measuring the current (*I*) from the CV curves and knowing the applied potential sweep rate (d*V*/d*t*) from the equation:

[9]
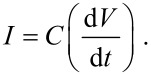


**Figure 54:**
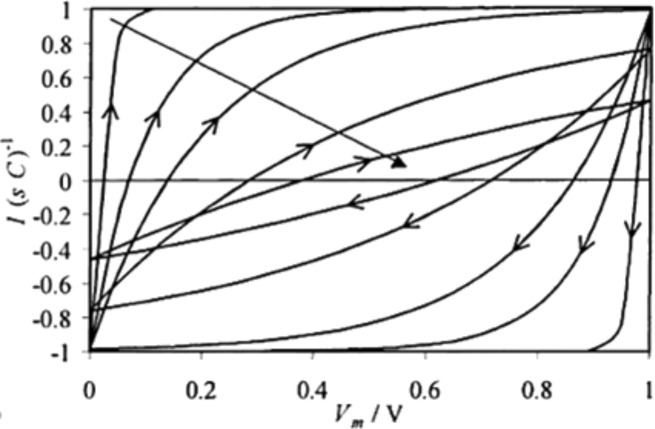
Simulation of CV curves with increasing internal resistance (1, 5, 10, 25 and 50 Ω) at 20 mV/s scan rate with *C* = 1 F and voltage range from 0 to 1 V. Reprinted with permission from [270]. Copyright (2001) Elsevier.

Most of the real CV curves for an EDLC show deviations from the ideal shape because of the electrolyte and electrode resistance and unwanted Faradaic reactions. For example, Figure 54 shows a simulation of CV curves with increasing internal resistance at a fixed scan rate [270].

**Figure 55:**
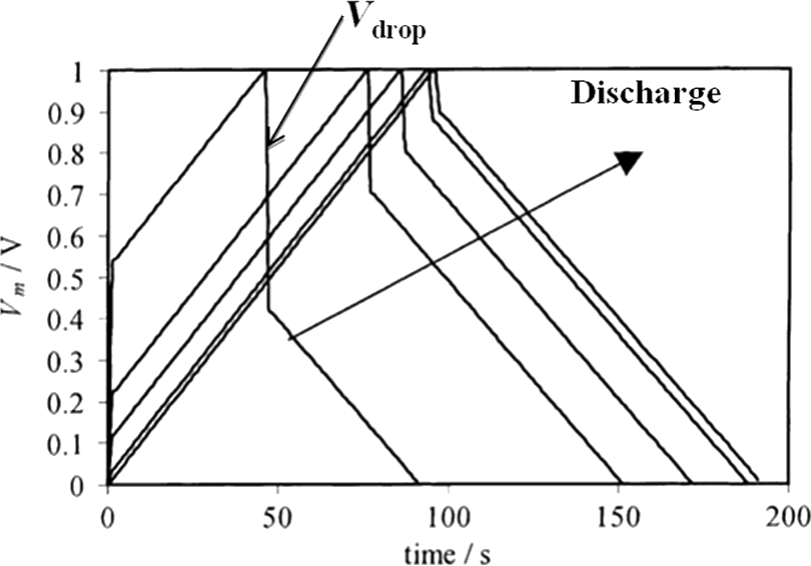
Simulation of the charge/discharge curves with increasing internal resistance (0, 1, 5, 10 and 25 Ω) at *I* = 10 mA. The smallest *V*_drop_ can be achieved with the smallest internal resistance. The longest total charging time is also achieved with the smallest internal resistance. Reprinted with permission from [270]. Copyright (2001) Elsevier.

The galvanostatic charge/discharge measurement is instead obtained by charging/discharging the supercapacitor at a certain defined current (*I*) within a certain voltage window. The galvanostatic charge/discharge measurement is plotted as time (s) vs voltage (V) (Figure 55). The capacitance and the internal resistance of the device can be extracted from this measurement technique. The capacitance is calculated from the slope of the charge or discharge curve with the equation [253]:

[10]
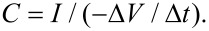


The effective series resistance (*R*_ESR_) is calculated from the voltage drop (*V*_drop_) that occurs at the initial portion of the discharge with the equation [253]:

[11]
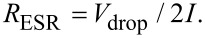


It can be seen in Figure 55 that the total charging time for a specific voltage limit decreases with increasing the internal resistance. This occurs because the electrode has a smaller effective charge capacity within a specific voltage window [270].

**Figure 56:**
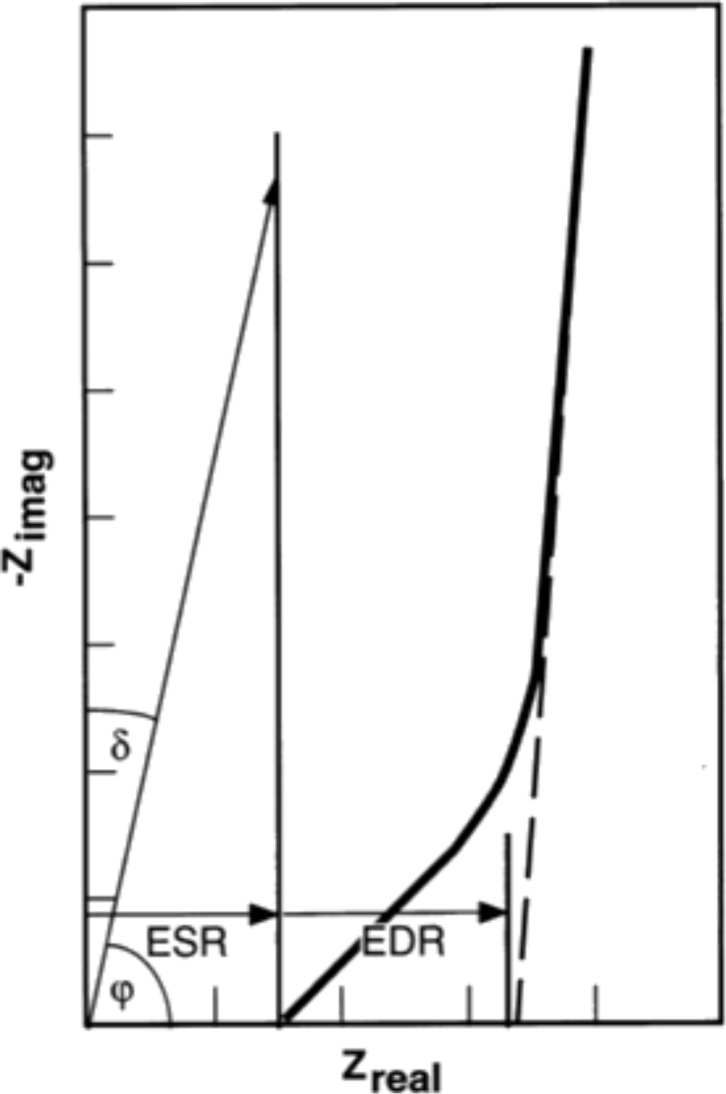
Schematic representation of the Nyquist impedance plot of an ideal capacitor (vertical thin line) and a supercapacitor with porous electrodes (thick line). Reprinted with permission from [266]. Copyright (2000) Elsevier.

Another useful measurement technique to reveal the properties of a supercapacitor is electrochemical impedance spectroscopy (EIS), which measures the impedance (*Z*) of a device over a range of frequencies. The data obtained are usually graphed as the real part of the impedance (*Z*_real_) vs the imaginary part of the impedance (*Z*_imag_), also called a Nyquist plot. Figure 56 shows a Nyquist plot of an ideal and a simplified supercapacitor. The ideal capacitor exhibits only a vertical line while a real one usually starts with a 45° line and then approaches a vertical line at higher frequencies. The 45° region, also called the Warburg region, is governed by the distributed resistance/capacitance in a porous electrode and by the electrolyte conductivity [253]. The effective series resistance (*R*_ESR_) and the equivalent distributed resistance (*R*_EDR_) can also be extracted from the Nyquist plot as shown in Figure 56 [266].

To determine the supercapacitor performance two other important factors, apart from the capacitance, need to be considered. One is the energy density that corresponds to the amount of energy stored per unit volume or mass and the other one is the power density that combines energy density with the speed that energy can be drawn out of a device.

The energy density per unit volume, expressed in Wh/cm^3^, is defined by the equation [253]:

[12]
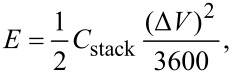


where *C*_stack_ = *C*/*V* is the volumetric stack capacitance expressed in F/cm^3^ and Δ*V* is the operating voltage window of the device.

The power density per unit volume, expressed in W/cm^3^ is defined by the equation [253]:

[13]
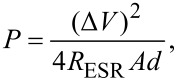


where *A* is the area of the electrodes, *d* is the distance between the electrodes, Δ*V* is the operating voltage window of the device and *R*_ESR_ is the total effective resistance of the device that can be extracted from the galvanostatic charge/discharge curves.

The maximum energy and power densities are achieved at the maximum voltage applied to the device, which is usually determined by the maximum voltage that the electrolyte can tolerate before decomposition and breakdown of the electrode material.

Using Equation 12, the easiest way to increase the energy density is to increase the capacitance (*C*) of the device, but this is not the only way. The energy density can also be improved by increasing the voltage window of the electrolyte (Δ*V*), which follows a quadratic law. Organic electrolytes can achieve higher voltages compared to aqueous electrolytes, allowing for a dramatic increase in the energy density. Unfortunately, they also show an effective series resistance (*R*_ESR_) of at least 20 times larger than the aqueous ones, which reduces the power density (Equation 13) [266].

In summary, several important characteristics of an EDLC have to be considered to maximize the performance of the device [253]:

- The specific surface area of the electrodes to increase the capacitance
- The conductivity of the electrodes to reduce the power density losses
- The resistance to any oxidation/reduction on the surface of the electrode to maintain good stability and performance
- Controlled size distribution of the pores that should match the size of the electrolyte ions
- Electrochemical stability of the electrolyte in the voltage operating window of the device
- Low interconnected resistance of the electrolyte
- Good wettability of the electrolyte on the electrode.

#### Carbon nanotubes and graphene in supercapacitors

Activated carbons (ACs) are the most commonly used materials for commercial electrodes in supercapacitors because of their stable electrical properties, large surface area and low cost. ACs can be produced by thermal and/or chemical activation of various types of materials containing carbon such as wood, coal, etc. For thermal activation, high temperatures are usually employed (from 700 to 1200 °C) in the presence of oxidizing gases. For chemical activation, the process temperatures are lower (from 400 to 700 °C) and require activating agents such as phosphoric acid, sodium hydroxide and others [265]. These two processes allow the production of a material with a high surface area (3000 m^2^/g) but with a wide pore size distribution consisting of macropores (>50 nm), mesopores (2–50 nm) and micropores (<2 nm) [271–272]. Even with such a high surface area, the experimental capacitance obtained with these materials is lower (<10 μF/cm^2^) when compared to the theoretical calculations [260]. This difference indicates that not all of the pores are contributing to the charge storage mechanism and that the specific surface area is not the only parameter to be considered in a supercapacitor [273]. Pore shape and structure, pore size distribution, electrical conductivity and wettability of the electrode are other important parameters that contribute to the performance of the device as previously discussed. The pore size distribution in ACs is still a problem to be addressed [265].

Carbon nanomaterials such as CNTs and graphene are excellent candidates to replace ACs as electrode materials in supercapacitors because of their remarkable chemical stability, large specific surface area, and high electric conductivity [274–275]. Commercial supercapacitors contain metal foils such as Al, Cu, and stainless steel, as current collectors which require special techniques to passivate the metal surface to avoid corrosion effects due to the use of alkali or acidic electrolytes [276]. Because of the high conductivity of CNTs and graphene, they can function as the capacitor electrode and the current collector, leading to a more simple and lightweight device.

**Figure 57:**
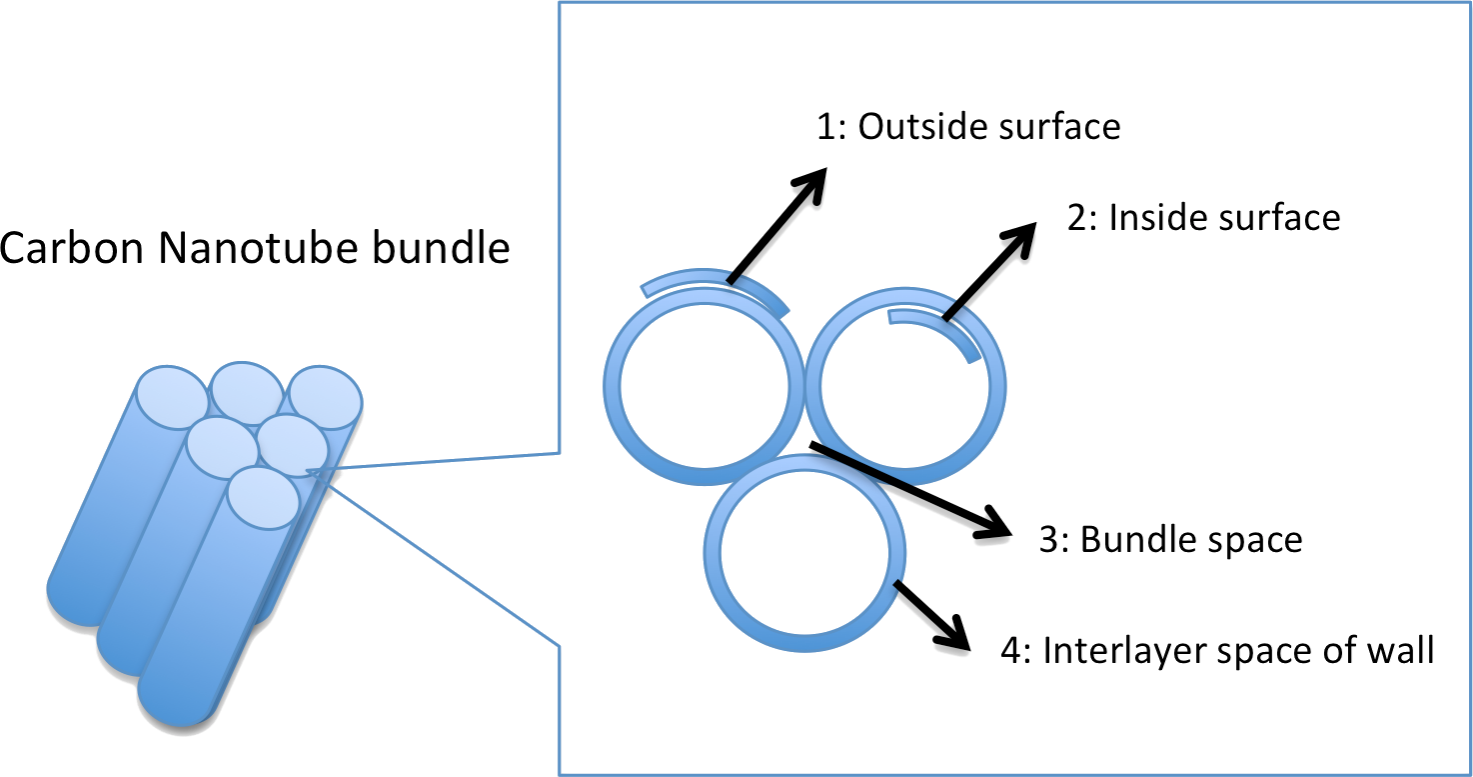
Schematic illustration of the space in a carbon nanotube bundle available for the storage of electrolyte ions.

CNTs in the form of either arrays grown on a substrate [277–278] or network films processed from a suspension [279] have been employed in supercapacitors. CNTs show a specific capacitance of 15–200 F/g [280] with a high power capability but a low energy density due to a small specific surface area (<500 m^2^ g^−1^) caused by the entangled arrangement of the CNTs with only the outermost tubes of the bundles exposed to the electrolyte [281] (Figure 57).

**Figure 58:**
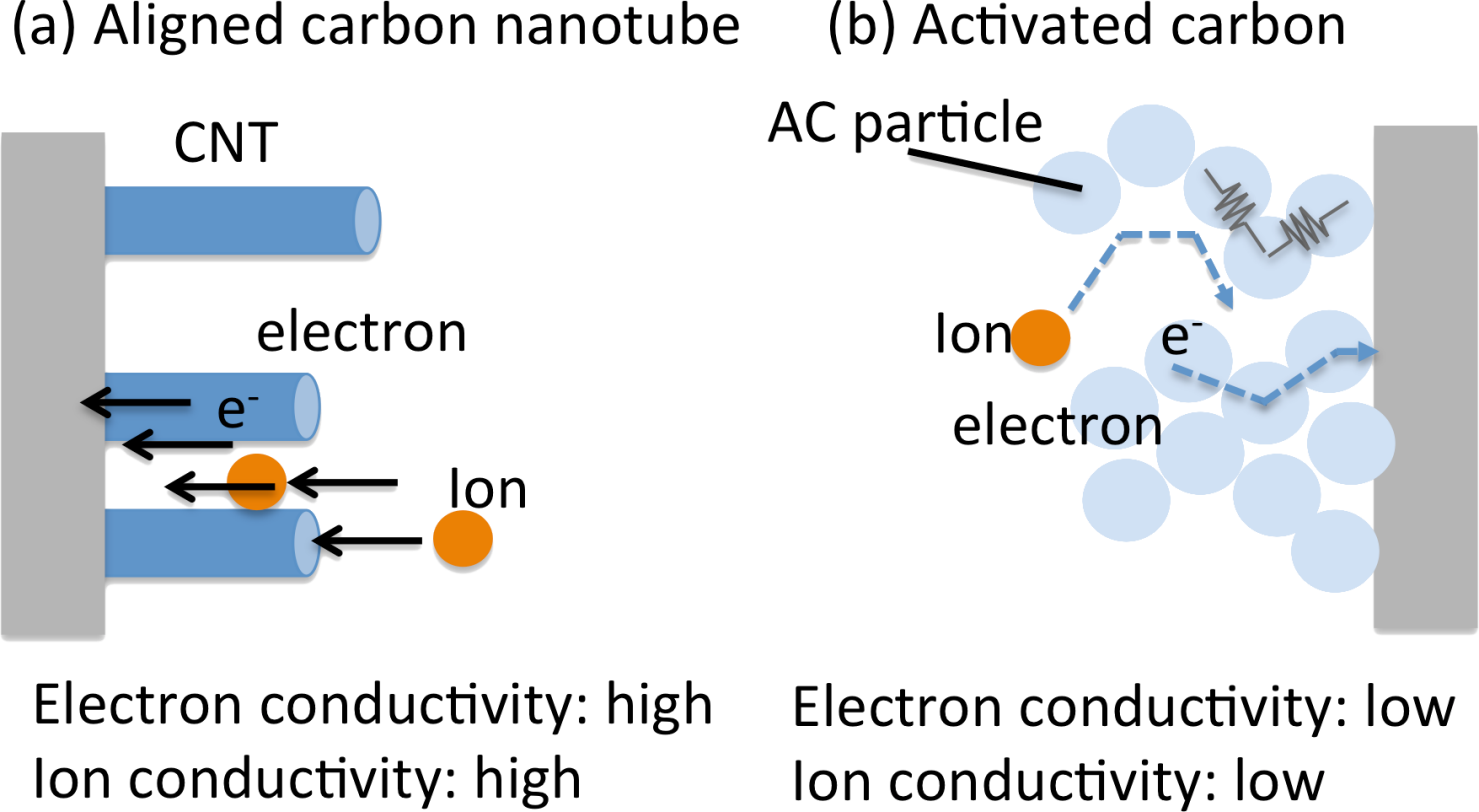
Comparison of conducting paths for electron and electrolyte ions in aligned carbon nanotubes and granular activated carbon.

CNTs grown on a substrate can be very useful for high power applications when compared to ACs. In fact, the schematic in Figure 58 shows that if the CNTs are all aligned on a current collector, the resistance can be low because the path for ions and electrons is much simpler than in the typical AC electrodes [281] .

MWNTs were the first to be proposed as an electrode for an EDLC, showing a capacitance of ≈100 F/g, a low surface area of ≈400 m^2^/g and a low power density value of 8 kW/kg [282]. However, apart from their high volumetric capacitance values [283] obtained by optimizing the growth process, SWNTs are preferred because they exhibit a higher surface area and consequently better overall performance [284].

Many strategies have been proposed to increase the surface area of SWNTs such as oxidizing methods, pyrolysis methods or by the use of liquid zipping effects [279,285–286]. The electrochemical oxidation in KOH can increase the capacitance by three times because it can facilitate the opening of some tubes, which increases the surface area. A “super growth” method has also been beneficial to increase the surface area to 1000 m^2^/g as compared to the 400–800 m^2^/g for commercial CNTs, proving that this material can potentially have better capacitive performance than ACs [286].

A combination of the “super growth” and oxidation methods has also been proposed in order to further increase the surface area to over 2000 m^2^/g with an energy density and power density of 24.7 Wh/kg and 98.9 kW/kg, respectively [287].

**Figure 59:**
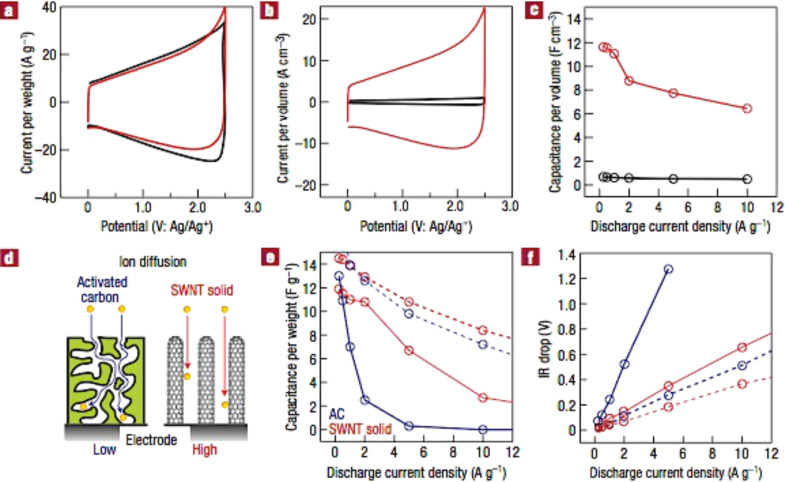
CV curves of the EDLC using the SWNT solid sheet (red) and as-grown forest (black) as electrodes, comparing the capacitance per weight (a) and capacitance per volume (b). (c) Change in the capacitance per volume using the SWNT solid sheet (red) and as-grown forest (black). (d) Schematic model comparing the ion diffusion for activated carbon and the SWNT solid material. (e) Capacitance versus discharge current density comparing SWNT solid (red) and activated carbon (blue) for 0.1 and 0.5 mm electrode thicknesses (dashed and solid lines, respectively). (f) Potential drop associated with an increase in internal resistance (IR drop) for SWNTs (solid, red) and activated carbon (blue) for 0.1 and 0.5 mm electrode thicknesses (dashed and solid lines, respectively). Reprinted with permission from [288]. Copyright (2006) Nature Publishing Group.

Vertical SWNTs with high purity and high density have also been grown by CVD and then removed from the substrate as a single unit, uniformly densified and engineered into different shapes by the zipping effects of liquid. The surface tension of the liquids and the strong van der Waals interactions can connect the SWNTs together in near-ideal graphitic spacing. With this method, no insulating binders were needed with a conductivity 20 times larger than ACs and a higher capacitive performance than in the conventional SWNT forest (Figure 59) [288].

**Figure 60:**
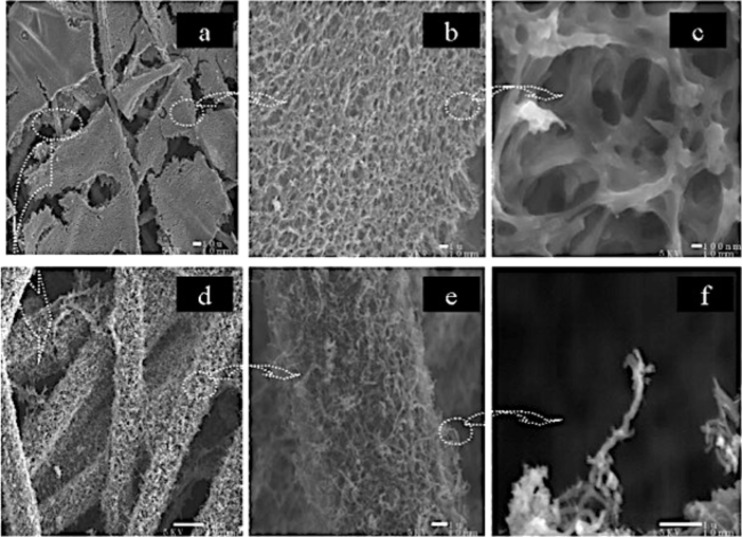
SEM images of CNT–carbon aerogel nanocomposites. Reprinted with permission from [289]. Copyright (2008) John Wiley and Sons.

Another alternative, a mixture of CNTs and carbon aerogel, has been proposed to increase the surface area to 1059 m^2^/g, obtaining a specific capacitance of 524 F/g (Figure 60) [289].

Recently, graphene and graphene derivatives have been considered for supercapacitor electrodes, not only for their exceptional electrical, thermal and mechanical properties previously described, but also for two other main reasons: the theoretical high surface area (2630 m^2^/g) [68] and the inexpensive methods of production [81]. In fact, for supercapacitor applications, methods like the chemical exfoliation of graphite or the thermal reduction of GO are probably the most used because of the straightforward, large production quantity of quality materials for electrodes. The addition of certain functional groups can also help to disperse the material in different solvents [290].

**Figure 61:**
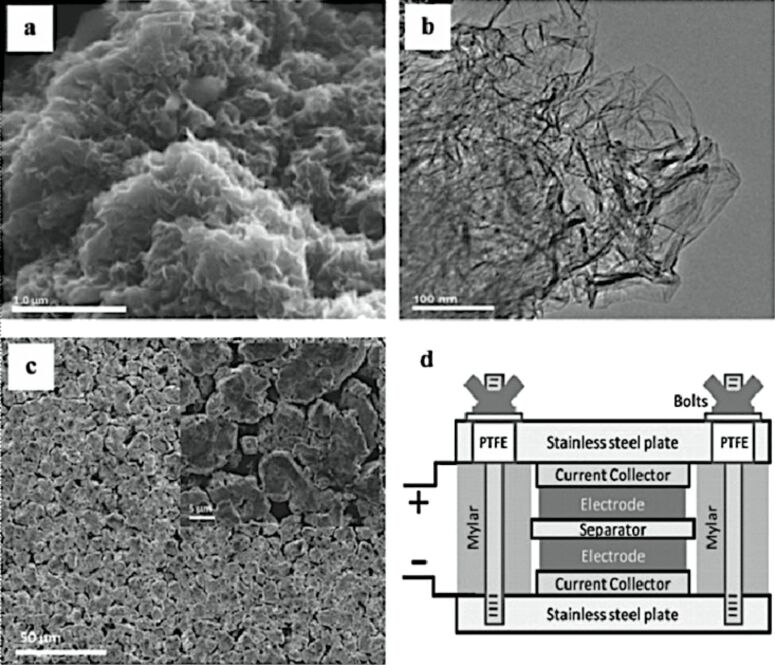
Graphene-based EDLCs utilizing chemically modified graphene as electrode materials. (a) Scanning electron microscopy (SEM) image of the material. (b) Transmission electron microscopy (TEM) image showing individual graphene sheets. (c) Low- and high- (inset) magnification SEM images of the electrode. (d) Schematic of test cell assembly. Reprinted with permission from [68]. Copyright (2008) American Chemical Society.

Stoller et al. [68] were the first group to explore the possibility of using chemically modified graphene, specifically reducing GO with hydrazine hydrate for EDLCs, obtaining specific capacitance values of 135 F/g and 99 F/g for aqueous and organic electrolytes, respectively, even with a relatively low surface area of 705 m^2^/g (Figure 61).

**Figure 62:**
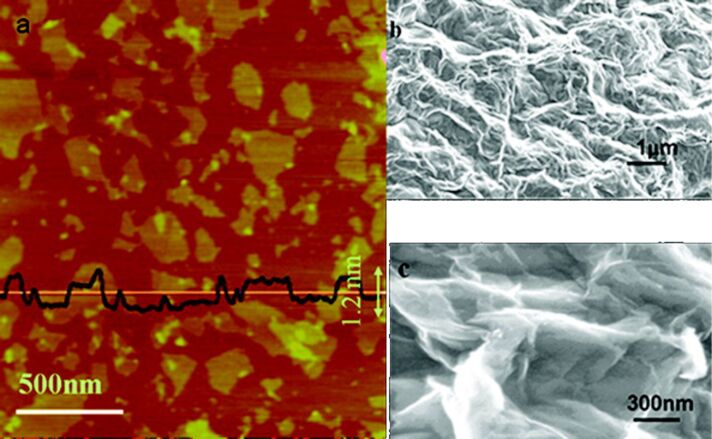
Morphology of graphene oxide and graphene-based materials. (a) Tapping-mode AFM image of graphene oxide and height profile plot. (b) and (c) SEM images at lower and higher magnification respectively of the graphene oxide reduced by a gas–solid reduction process. Reprinted with permission from [291]. Copyright (2009) American Chemical Society.

In order to maximize the performance by increasing the surface area, Wang et al. [291] proposed to reduce the GO in a gas–solid reduction process (Figure 62). In this way, they were able to obtain a capacitance of 205 F/g with a power density and energy density of 10 kW/kg and 28.5 Wh/kg, respectively.

Others, like Chen et al. [292], proposed to mildly reduce the GO with hydrobromic acid in order to maintain some oxygen functional groups that could promote the wettability of the electrode and avoid restacking of the graphene sheets, allowing better penetration of the electrolyte ions through the film. With this method, they were able to achieve a very high capacitance value of 348 F/g.

A GO exfoliation process was also explored as a method to fabricate EDLC electrodes. In particular, Lv et al. [293] showed that low temperature exfoliation of GO (200 °C) led to graphene-based electrodes with 264 F/g and 122 F/g in aqueous and organic electrolytes, respectively.

Instead, Zhu et al. [122] proposed a very inexpensive way to reduce GO for EDLC electrodes. They reduced the material in a conventional microwave oven obtaining a surface area of 463 m^2^/g with a specific capacitance of 191 F/g [122]. Certainly, these are not the highest values reported but the ability to use a simple microwave creates a pathway for a scalable and inexpensive process to fabricate electrodes for supercapacitors.

**Figure 63:**
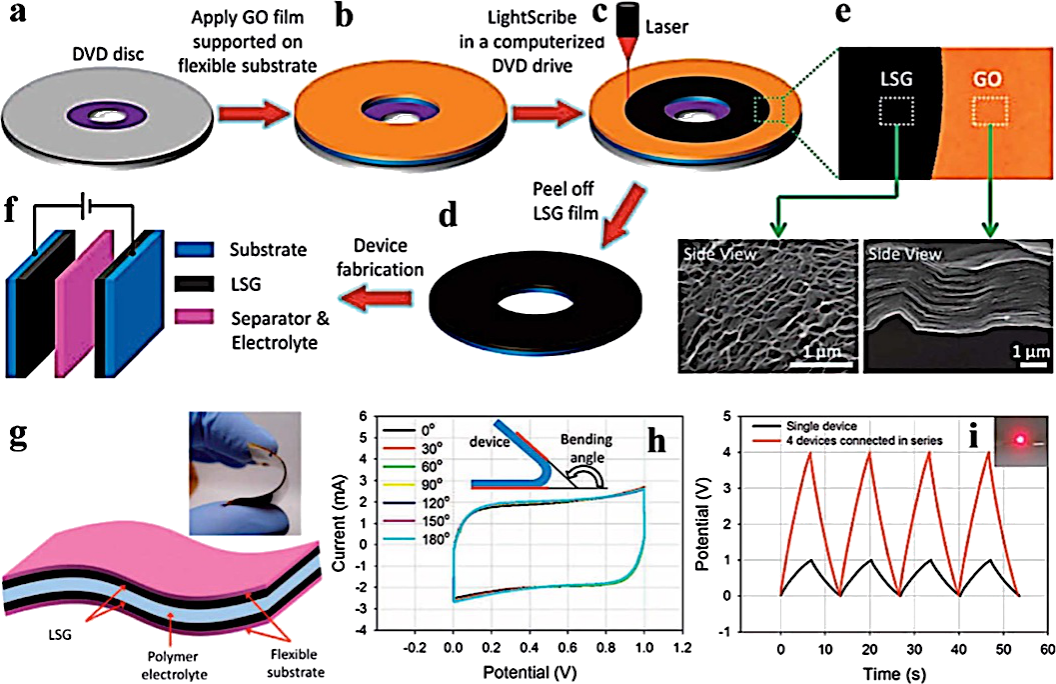
(a–d) Schematic illustration of the process to make laser-scribed graphene-based electrochemical capacitors. A GO film supported on a flexible substrate is placed on top of a LightScribe-enabled DVD media disc, and a computer image is then laser-irradiated on the GO film in a LightScribe DVD drive. (e) As shown in the photograph, the GO film changes from golden brown to black as it reduced to laser-scribed graphene. The low power, infrared laser changes the stacked GO sheets immediately into a well-exfoliated few-layered laser-scribed film, as shown in the cross-sectional SEM images. (f) A symmetric supercapacitor is constructed from two identical laser-scribed electrodes, an ion-porous separator, and an electrolyte. (g) A schematic diagram of the all-solid-state device illustrates that the gelled electrolyte can serve as both the electrolyte and separator. The inset is a digital photograph showing the flexibility of the device. (h) CV curves collected at a scan rate of 1000 mV/s when the device was bent at different angles. (i) Galvanostatic charge–discharge curves for four devices connected in series. The inset image shows the glow of an LED light by the four devices in series. Reprinted with permission from [294]. Copyright (2012) The American Association for the Advancement of Science.

A similar concept was proposed in 2012 by El-Kady et al. [294] using a standard LightScribe DVD optical drive to reduce GO with a gel electrolyte based on poly(vinyl alcohol) (PVA)-H_3_PO_4_ to fabricate flexible devices (Figure 63). The material showed a high surface area of 1520 m^2^/g and a specific capacitance of 265 F/g.

**Figure 64:**
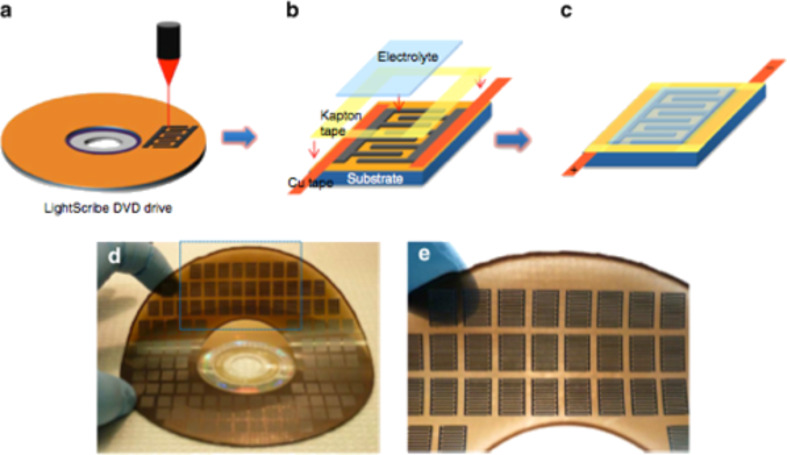
(a–c) Schematic diagram showing the fabrication process for a laser-scribed graphene micro-supercapacitor. A GO film supported on a PET sheet is placed on a DVD media disc. The disc is inserted into a LightScribe DVD drive and a computer-designed circuit is etched onto the film. The laser inside the drive converts the golden brown GO into black, laser-scribed graphene at precise locations to produce interdigitated graphene circuits (a). Copper tape is applied along the edges to improve the electrical contacts, and the interdigitated area is defined by polyimide (Kapton) tape (b). An electrolyte overcoat is then added to create a planar micro-supercapacitor (c). This technique has the potential for the direct writing of microdevices with a high areal density (d,e). More than 100 microdevices can be produced on a single run. The microdevices are flexible and can be produced on virtually any substrate. Reprinted with permission from [295]. Copyright (2013) Nature Publishing Group.

In 2013, El-Kady et al. [295] were able to further develop this technique by fabricating more than 100 micro-supercapacitors on a single DVD disc in 30 min. The devices were built on flexible substrates to be integrated with MEMS or CMOS technologies in a single chip (Figure 64). These micro-supercapacitors demonstrated a very high power density of 200 W/cm^3^.

Planar structures have limited capacitance due to the restacking of graphene sheets, which reduces the surface area. Therefore, other graphene structures have been proposed to further boost the performance of EDLCs. Porous, 3D graphene networks have been synthetized by freezing and drying a chemically reduced GO dispersion [296–297] or by CVD on nickel foam [298–299] to overcome the limitations of planar structures.

**Figure 65:**
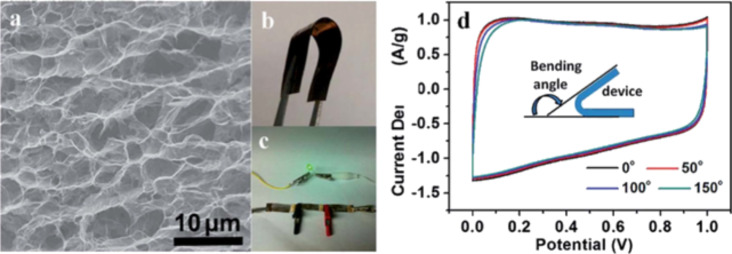
(a) SEM image of the interior microstructure of a graphene hydrogel. (b) Photograph of the flexible solid-state supercapacitor based on a graphene hydrogel film. (c) Photograph of a green LED powered by the three supercapacitors in series. (d) CV curves of the flexible solid-state device at 10 mV/s for different bending angles. Reprinted with permission from [296]. Copyright (2013) American Chemical Society.

Xu et al. [296] prepared a 3D, porous, reduced, GO hydrogel with a capacitance of 186 F/g and a capacitance decay of only 8.4% after 10,000 charge/discharge cycles in a PVA-H_3_PO_4_ gel polymer electrolyte (Figure 65). Even with such a high capacitance, the power density was limited to 0.5–5 kW/kg, indicating that the porous structure has a large internal resistance and needs to be combined with a Au current collector. An electrode that also functions as a bendable, high efficiency current collector is usually preferred for supercapacitor applications in order to maintain a high power density value [300–301].

**Figure 66:**
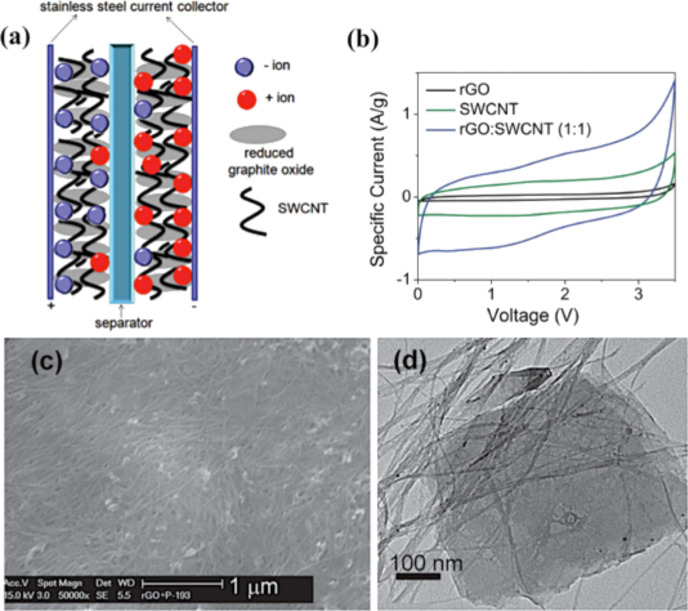
(a) Schematic illustration of a supercapacitor cell fabricated from reduced graphite oxide (rGO) and SWNTs. (b) CV curves of the materials at 10 mV/s. (c) SEM image of the hybrid material consisting of rGO and SWNTs in a 1:1 weight ratio. (d) TEM image of the hybrid material showing few-layer graphene sheets covering a network of SWNTs. Reprinted with permission from [304]. Copyright (2012) John Wiley and Sons.

Hybrid structures of CNTs and graphene electrodes have also been proposed for supercapacitors in order to combine the properties of both materials, boosting the capacitance and the energy density of the devices [302–306]. In fact, the idea is to increase the surface area and have a defined architecture for the electric transport by using graphene to store the charge and CNTs as an efficient charge transport material. Jha et al. [304] demonstrated that a device made of reduced GO mixed in a proportion of 1:1 with SWNTs achieved a specific capacitance of 222 F/g and with an energy density of 94 Wh/kg in an ionic liquid electrolyte (Figure 66).

**Figure 67:**
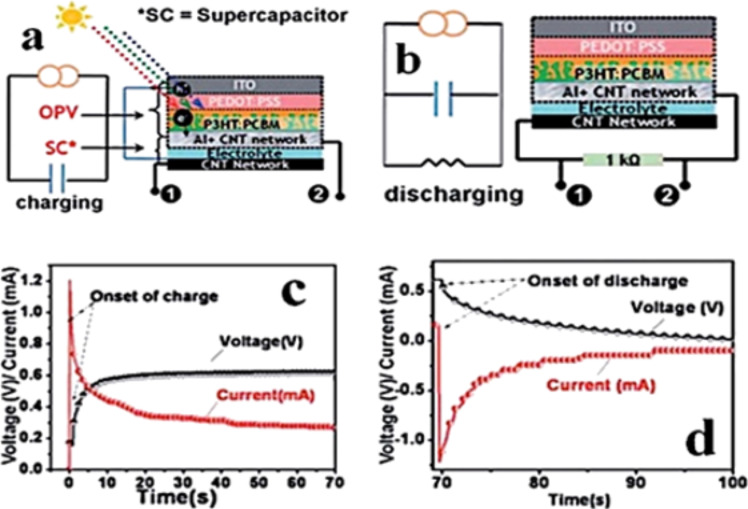
(a) and (b) are schematic, equivalent circuit illustrations for a polymer solar cell and a supercapacitor during charging and discharging, respectively. The voltage and current profiles versus time for the charging (c) and discharging (d) process. Reprinted with permission from [307]. Copyright (2010) Royal Society of Chemistry.

Flexible supercapacitors have been integrated into organic solar cells in order to eliminate the energy loss in the wiring between the energy conversion device and the storage device. Wee et al. [307] demonstrated that it is possible to integrate both devices in a single, printable, all-solid device. The supercapacitor was charged by a polymer solar cell and the discharge was achieved by connecting it to a resistor (Figure 67). The capacitance obtained from the discharge was only 28 F/g but the possibility to combine both devices onto a flexible and printable surface opens up the avenue to a scalable and cheap process of a simultaneous generation and storage device.

## Conclusions

The demand for energy is rapidly increasing due to dramatic population growth and technological advances worldwide. Conventional energy sources are limited and pollute the environment. Photovoltaics represent a viable solution to produce clean energy but its costs is still high due to the materials and process techniques involved. Moreover, an efficient energy storage system is required to be independent from the grid, because the sun is an intermittent energy source. Carbon, one of the most abundant materials found on earth, can be a valid material for both energy generation and storage. As was outlined in this review, it can be employed in real-world devices such as organic solar cells and supercapacitors in the form of one or more of its allotrope forms (e.g., graphene, carbon nanotubes, fullerenes) by employing inexpensive synthesis and process methods based on printing and roll-to-roll techniques.

In this paper, different approaches to synthesize and employ carbon nanomaterials for energy generation and storage applications have been explored. Carbon nanomaterials used for organic solar cells have been thoroughly reviewed. Graphene produced by electrochemical exfoliation was discussed as a viable solution to produce a large quantity of transparent electrodes for organic solar cells. Achieving a high quality material on a large scale still remains an issue in order to compete with conventional conducting transparent electrodes such as ITO. However, its potential for production on flexible substrates makes it very appealing for the organic solar cell field where roll-to-roll techniques have been recently employed to increase the production volume. Additionally, fullerene derivatives, CNTs and graphene oxide could help to boost the performance of organic solar cell devices if employed in the active or buffer layers. In fact, their semiconducting properties can be tuned by doping with other materials or by changing their physical structure in order to absorb a broader range of solar spectrum wavelengths.

Carbon nanomaterials, in particular carbon nanotubes and graphene, have also been proven to be very efficient and reliable materials for energy storage. The high specific surface area and conductivity of graphene are two key features for employing this material in supercapacitors. The ability to use a solid-state electrolyte composed of graphene oxide or a gel polymer electrolyte allow for the possibility of printable, flexible devices that do not require encapsulation. Although carbon nanotubes generally have a relatively low specific surface area, they can still be employed in combination with graphene to increase the conductivity of the electrode or the surface roughness of the film, resulting in an increase in the number of ions stored at the electrode/electrolyte interface.

Carbon continues to surprise researchers with its extraordinary properties. Completely new carbon structures have been synthesized over the last 20 years, from 0D fullerenes to 1D nanotubes and 2D graphene. The low cost of this element, the sixth most abundant element on earth, makes it an attractive choice to replace conventional materials for energy generation and storage applications.
